# Assessment of the Prevalence of Alcoholic Beverage Consumption and Knowledge of the Impact of Alcohol on Health in a Group of Polish Young Adults Aged 18–35: A Cross-Sectional Study

**DOI:** 10.3390/ijerph192315425

**Published:** 2022-11-22

**Authors:** Martyna Wysokińska, Aleksandra Kołota

**Affiliations:** Department of Dietetics, Institute of Human Nutrition Sciences, Warsaw University of Life Sciences (SGGW-WULS), 159c Nowoursynowska Street, 02-776 Warsaw, Poland

**Keywords:** alcohol, alcohol consumption, young adult, knowledge, public health, health promotion

## Abstract

Alcoholic beverages are widely consumed worldwide, especially by young adults. Their excessive consumption is associated with numerous health, social and financial damages. The level of knowledge of young adults about the health effects of consuming alcoholic beverages is low, and research in this area is conducted on small, unrepresentative groups. This cross-sectional study aimed to assess the prevalence of alcoholic beverage consumption and the level of knowledge about the impact of ethyl alcohol on health in a group of people aged 18–35. The survey results indicate that the majority of respondents regularly consume alcoholic beverages (94.6%), and they are at a low risk of excessive consumption (*p* < 0.0001). The most frequently chosen alcoholic beverage in the studied group was beer, and the least chosen one was vodka. The main motive for reaching for alcoholic beverages was the desire to improve mood. Respondents did not indicate significant changes in alcohol consumption during the COVID-19 pandemic, but participants in the high-risk group more often indicated an increase in alcohol consumption (*p* = 0.0025). The analysis of the level of knowledge showed that the participants in the study had an average or low level of knowledge about the effects of ethanol on health, with no significant relationships between the study groups. The obtained results indicate a strong need for the continuous education of young people on the effects of the excessive consumption of alcoholic beverages on the body, with particular emphasis on the consequences of using alcohol as a mood-enhancing agent.

## 1. Introduction

Alcoholic beverages are the most frequently used psychoactive agent [1], and their regular consumption is declared by approximately 2.3 billion people in the world, with the average consumption in the world in 2019 estimated at 5.8 litres of pure ethanol per person over the age of 15 years [2].

The consumption of alcoholic beverages varies depending on the country, culture, and age of the drinkers [3]. Soundararajan et al. [4] indicate that young adults consume twice as much ethyl alcohol as adults per month. Although the consumption of alcoholic beverages is increasing more and more among the elderly, young adults are much more likely to engage in heavy binge drinking than people from other age groups [3,4]. The first exposure to ethanol is observed in increasingly younger age groups [5], and early alcohol initiation is associated with an increased risk of alcohol abuse later in life, as well as an increased risk of alcohol-related injuries [4,5,6]. Moreover, it has been shown that with age, the amount and intensity of alcohol consumption decrease [3].

The results obtained by Visontay et al. [7] indicate that harmful alcohol consumption by young adults may have decreased between 1989 and 2015. This means that alcohol-related health problems in this age group may decrease despite persistent addiction problems and short- and long-term health consequences. Despite the promising results, the authors emphasise the need for further research, as it is not known whether the significant reduction in alcohol consumption in young adults will be sustained later in life. In addition, it is also important to identify the cause of the observed decrease in harmful alcohol consumption by young adults. Burgess et al. [8] suggest that this change is generational testifying to the greater sense of young people, and Torronen et al. [9] suggest that it may be related, inter alia, with the increased use of social media, but also a greater interest in a healthy lifestyle and physical activity. Given that drinking alcohol is firmly embedded in many cultures, Irizar et al. [10] recommend focusing health policy on changing the current drinking culture by establishing publicly understandable drinking guidelines and increasing the availability of non-alcohol alternatives.

The COVID-19 pandemic has recently been an additional factor influencing the consumption of alcoholic beverages. According to estimates, before the COVID-19 pandemic, nearly one in three adults in OECD (Organization for Economic Cooperation and Development) countries used to become inebriated at least once a month, but pandemic-related restrictions have impacted both alcohol consumption habits and sales of alcoholic beverages [11]. The survey data indicate not only an increase in the amount, but also the frequency of consumption of alcoholic beverages [11], as well as an increase in the frequency of heavy drinking during the pandemic compared to the period before the pandemic [12,13]. Research conducted in Poland showed that over 30% of respondents changed the frequency of consumption of alcoholic beverages during the pandemic, with 14% reporting an increase in their consumption [14]; similar results were obtained by German researchers who noted an increase in alcohol consumption in 14% of young adults and 17% of adults [15]. On the other hand, a survey conducted in Canada showed that 18% of respondents who were more often at home due to coronavirus declared that their alcohol consumption had increased [16]. The recent literature indicates that during the COVID-19 pandemic, in young adults the greatest increase in alcohol consumption was associated with the severity of symptoms of depression [17]. Other authors note that the changes in the frequency of alcohol consumption were likely partly due to drinking alone and at home, which was exacerbated by the COVID-19 pandemic [18]. For this reason, specialists dealing with the prevention of risky behaviours have proposed the implementation of comprehensive measures to promote the reduction of alcohol consumption in order to cope with stress and isolation [19,20]. However, the latest research results suggest that in young adults, the use of a mobile application to target alcohol-related behaviour may be more effective than traditional interventions [21].

Recognising the problem of the growing consumption of alcoholic beverages, the World Health Organization emphasises that alcohol consumption during a pandemic may potentially exacerbate health problems and engagement in risky behaviour [22]. Ethyl alcohol, as a component of alcoholic beverages and a psychoactive and toxic substance, is one of the most common risk factors for many diseases, as well as premature death [23]. Long-term alcohol consumption increases the incidence of severe mental health problems, including severe depression and anxiety disorders. The consumption of ethyl alcohol by young people is also positively correlated with deliberately inflicted injuries, such as self-mutilation and interpersonal violence, as well as unintentional injuries, the most frequent of which are road accidents, poisoning, falls, fires and drowning [23,24,25,26]. Ethanol also reduces inhibitions and increases impulsivity, which may directly translate into aggressive behaviour after drinking alcoholic beverages, which is common among young people [27].

Despite the undoubtedly adverse effects of the excessive consumption of alcoholic beverages on health, knowledge of the consequences of consuming alcoholic beverages or the impact of ethanol on health seems to be still insufficient [28,29]. The studies published so far are often conducted with the participation of a small group of respondents who cannot be considered representative [30,31]. The research on the knowledge of young adults carried out in Poland showed that people who consumed alcoholic beverages in a risky manner were characterised by a low level of knowledge about the negative effects of alcohol abuse [32,33], and in addition, general awareness of the symptoms of alcohol dependence in the Polish population is very low [34]. Analysing research conducted in other countries, it was observed that the general knowledge of the British regarding alcoholic beverages is low, because most of the surveyed people did not know the concept of a “standard drink” in the context of ethyl alcohol, were unable to correctly estimate its energy value or indicate the possible dangers resulting from its excessive consumption [28,29].

### Research Aim and Hypothesis

Considering that ethyl alcohol is often abused by young adults and their knowledge of the effects of ethanol on health is insufficiently researched, the aim of this study was to assess the frequency, quality and style of alcohol consumption by young adults living in Poland aged 18–35 and to analyse the level of knowledge of the impact of alcoholic beverages on health.

During the study, a research hypothesis was put forward, according to which young adults aged 18–35 drink in a risky manner, and their knowledge about the influence of ethanol on human health is low.

## 2. Materials and Methods

### 2.1. Ethical Statement

Due to the epidemic situation in the country, the study was conducted using an anonymous online questionnaire hosted on the Google platform. All procedures were approved by the Ethics Committee of the Faculty of Human Nutrition Sciences of the Warsaw University of Life Sciences, Poland (No 3/2022). All participants consented to participate in the study and had the option to withdraw at any stage of the study.

### 2.2. Studied Group

The cross-sectional study was conducted in Poland in a group of adults aged 18–35. The data for the research was collected using the snowball method [35] over two time periods. The first round of survey data collection took place in the period from June to September 2021. Then, due to the increase in frequency of the consumption of alcoholic beverages observed in the winter [36], it was decided to return to collecting data from the respondents in the period from March to April 2022.

**Figure 1:**
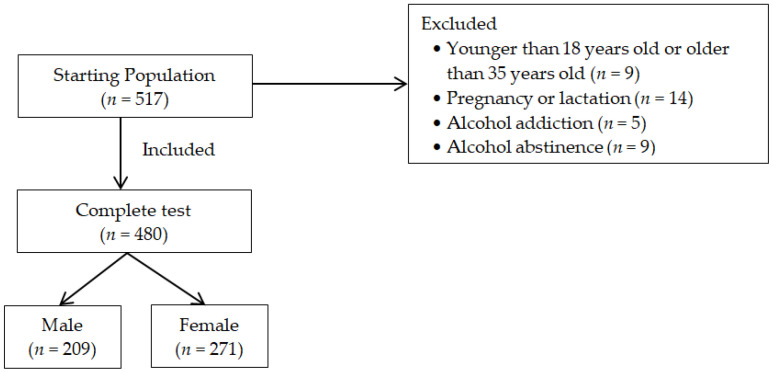
The flowchart showing the starting population, the exclusion criteria and the final population that completed the study.

In the study 517 respondents participated. Of these, 1.8% (*n* = 9) were excluded due to age and 2.7% (*n* = 14) were excluded due to pregnancy or lactation. Additionally, 1.7% (*n* = 9) of the participants declared complete abstinence and 1% (*n* = 5) were addicted to alcohol, which resulted in their exclusion from the study. In the end, the data included in this study concerned the analysis of questionnaires that were correctly completed by 480 participants (Figure 1).

### 2.3. Applied Questionnaire

The questionnaire created for this study was divided into three main parts (Table S1). The first part included questions about gender, age, body weight, height, marital status, size of the city of residence, employment and health assessment. Moreover, female respondents were asked about their current physiological state (pregnancy or lactation). Due to the risk associated with the consumption of alcoholic beverages by pregnant and breastfeeding women [37,38], as well as the recommended total abstinence in these periods [39], pregnant or breastfeeding women were excluded from the study. The second part included questions about the frequency and style of alcohol consumption, and the third focused on the assessment of the respondents’ knowledge of the impact of alcohol on health.

Questions on the frequency of consumption of alcoholic beverages were developed on the basis of the Chodkiewicz [40] and Sierosławski [41] questionnaires and the questionnaire published by Pretendent [42]. Most of the survey questions were closed, with the exception of age, weight, height, allergies and chronic disease. Moreover, the questionnaire included questions for which it was possible to provide one’s own answer, defined as “other”, if the variants proposed by the authors of the questionnaire were not appropriate. In the question “What type of alcoholic beverages do you most often consume currently?” the respondents had the opportunity to provide a type of alcoholic drink from outside the list proposed in the questionnaire. Single, non-recurring responses were classified as “other”. The section assessing drinking style used the Alcohol Use Disorder Identification Test (AUDIT) [43]. This test is a brief self-reported alcohol-screening test that is effective for assessing unhealthy alcohol use [43]. This instrument is a 10-question survey with a total score ranging from 0 to 40 points. Each item is rated from 0 to 4 points. A score of 8 or more on the AUDIT could indicate people who are risky drinkers or have alcohol use disorders. Commonly, the likelihood of a person having an alcohol use disorder is directly proportional to the highest score on the test. The second questionnaire whose questions were used in this part was developed by Żołnierczuk-Kieliszek et al. [44]. The last part, concerning the respondents’ knowledge of the impact of ethyl alcohol on health, was prepared on the basis of the Chodkiewicz questionnaire [40], Knowledge of Psychiatric Aspects of Alcohol Questionnaire [45] and the questionnaire published by the Health Research Board [46]. In order to estimate the level of respondents’ knowledge, a knowledge index developed for the purpose of this study was used. This instrument is a 28-question survey with a total score ranging from 0 to 28 points (Table S2). Each item is rated as 0 or 1 point. The answers to the questions considered “correct” are based on the current literature [47,48,49,50,51,52,53,54,55,56,57,58,59,60]. For three questions, 1 point was given to each respondent, regardless of the answer given, due to different interpretations. The interpretation of the Knowledge Index Score is presented in Table 1. In addition, the questionnaire also included a question about the impact of the COVID-19 pandemic on changes in drinking habits. In the question about sources of knowledge on the impact of ethanol on health, the answers provided by the authors of the questionnaire were “own observation”, which meant the individual experiences and observations of the respondent and “peers”, which meant learning about the impact of ethanol on health from the experiences of peers.

### 2.4. Statistical Analysis

The required minimum sample size was calculated at 384 respondents. The sample size was calculated for the Polish population aged 18–35 (in total 8,267,480, based on Central Statistical Office data [61] in Poland), with a 95% confidence level and a 5% margin of error. A percentage of 50% was adopted, which maximizes the sample size (due to the lack of data on the expected percentage of results). Therefore, the collected sample of 480 respondents was interpreted as sufficient. 

The statistical analysis was conducted using Statistica, version 13.0 (Statsoft Inc., Tulsa, OK, USA). To check the internal reliability of the questionnaire for the analysed group, Cronbach’s alpha coefficient (Table S3) was used, which was at a respectable level, indicating good internal consistency. The Kolmogorov–Smirnov test with Lillefors correction was used to analyse the normality of the distribution of continuous results. Then, due to non-parametric distributions, the subgroups were compared using Pearson’s chi-square test (χ^2^) and correlation analysis, which was performed using Spearman’s rank correlation coefficient. The significance level was set at *p* < 0.05.

## 3. Results

### 3.1. Characteristics of the Studied Group and Results of the AUDIT Test

The characteristics of the anthropometric features of the participants included in the study are presented in Table 2. Women constituted more than half of the respondents, and statistical analysis showed that women had a significantly lower body mass index (BMI) than men participating in the study (*p* < 0.0001), although all respondents had a normal body weight.

Table 3 presents the detailed characteristics of the socio-demographic features of the studied group. The men participating in the study worked part-time significantly more often compared to the women, the majority of whom were students (*p* < 0.0001).

Table 4 presents the results of the AUDIT test, which enables the assessment of alcohol consumption habits. The statistical analysis showed that significantly more participants in the studied group were characterised by a low risk level of alcohol consumption (66.7% vs. 33.3%, *p* < 0.0001). It has been shown (Table S4) that, compared to participants with a low risk level alcohol consumption, a significantly higher percentage of participants with a risky drinking style declared drinking alcohol 2–3 times a week (35.0% vs. 11.9%, *p* < 0.0001), a significantly lower percentage declared consuming six or more alcohol drinks on one occasion less than once a month (55.6% vs. 31.3%, *p* < 0.0001). In addition, it can be noted that a lower percentage of hazardous drinkers during the last year have been able to remember what happened the night before, despite drinking (89.1% vs. 28.1%, *p* < 0.0001), have never done something inappropriate (90.0% vs. 34.4%, *p* < 0.0001), have never had problem with stopping drinking after starting (96.6% vs. 51.9%, *p* < 0.0001), have never had to drink in the morning to recover from “heavy drinking” (97.2% vs. 53.1%, *p* < 0.0001), have never experienced guilt or remorse (77.5% vs. 23.8%, *p* < 0.0001). Moreover, a higher percentage of respondents with a low level of risky drinking style declared that during the last year no one had been injured as a result of drinking alcohol (90.6% vs. 56.3%, *p* < 0.0001), and no one had had an interest in or suggested restricting alcohol consumption (95.9% vs. 63.8%, *p* < 0.0001). Additional analyses assessing the impact of the data collection period on the audit test results are included in the Supplementary Materials (Tables S11 and S12). No influence of the participants’ recruitment period on the risk of alcohol consumption was found (*p* > 0.05), however, a significantly higher percentage of respondents recruited in the second period of data collection declared that they did not consume alcoholic beverages (8.5% vs. 0.0%, *p* = 0.0050). Furthermore, participants recruited earlier significantly more frequently consumed seven or more drinks on one occasion (16.3% vs. 10.3%, *p* = 0.0067). Moreover, the same group of study participants significantly less frequently declared problems with stopping drinking after starting (2.1% vs. 10.6%, *p* = 0.0187) or met with comments from relatives about their excessive drinking (9.4% vs. 3.5%, *p* = 0.0355).

### 3.2. Analysis of the Correlation between Alcohol Consumption and Results of the AUDIT Test as Well as the Knowledge Test in the Studied Group

The analysis of the correlation between age and place of alcohol initiation, the type of alcoholic beverages consumed first, the type of alcoholic beverages consumed most often, changes in the consumption of alcoholic beverages due to the COVID-19 pandemic, the amount of expenditure on the purchase of alcoholic beverages and the frequency of binge drinking, and the results of the AUDIT test and the knowledge test in the group of young adults who participated in the study are presented in Table 5. It was observed that the analysed indicators were not related to the results of the knowledge test (*p* > 0.05) except for the place of alcohol initiation, where a negative correlation was observed (*p* = 0.0190; R = −0.1071), but some associations were observed for the AUDIT test results. Positive correlations were observed for the type of alcoholic beverages consumed most frequently (*p* < 0.0001; R = 0.1830), the amount of money spent on purchasing alcoholic beverages (*p* < 0.0001; R = 0.2395), and the frequency of binge drinking (*p* < 0.0001; R = 0.3485), and negative correlations were observed for the age of alcohol initiation (*p* = 0.0001; R = −0.0975) and the type of alcoholic beverage consumed first (*p* = 0.0327; R = −0.1730). Statistical analysis showed (Table S5) that a significantly higher percentage of respondents declaring their first contact with ethyl alcohol at the age of 10–15 were people who consume ethyl alcohol at a risky level (27.5% vs. 46.3%, *p* < 0.0001). Moreover, compared to people with a low risk level of alcohol consumption, these people significantly more often, consumed alcoholic beverages at a social event for the first time, and vodka was the first alcoholic beverage consumed more often (39.4% vs. 23.8%, *p* = 0.0344 and 23.8% vs. 15.9%, *p* = 0.0214, respectively). Additionally, statistical analysis showed (Table S6) that people with a risky style of drinking alcohol, compared to people with a low risk level of alcohol consumption, choose vodka significantly more often (20.0% vs. 5.3%, *p* = 0.0001), as well as spending significantly more money (over 24 USD) on buying alcohol (26.3% vs. 4.7%, *p* < 0.0001). They also become inebriated more often (61.9% vs. 21.3%, *p* < 0.0001), and declared that they drunk more alcohol during the COVID-19 pandemic than before the pandemic (18.1% vs. 7.8%, *p* = 0.0025). Additional analyses assessing the impact of the data collection period on the alcohol consumption habits are included in the Supplementary Materials (Tables S13 and S14). No significant relationships were found in the discussed parameters, except for the amount of money spent on alcoholic beverages. Participants recruited to the study in the first period of data collection significantly more often spent much more money on alcoholic beverages (34.1% vs. 28.6%, *p* = 0.0148).

The analysis of the correlation between the frequency of consumption of various types of alcoholic beverages and the results of the AUDIT test, as well as the results of the knowledge test is presented in Table 6. It was observed that the frequency of consumption of various types of alcoholic beverages was not related to the results of the knowledge test (*p* > 0.05), but some relationships were observed for the results of the AUDIT test. Positive correlations were observed for the frequency of consumption of “coloured” alcoholic beverages in the 12 months prior to the study (*p* < 0.0001; R = 0.4153), as well as for the frequency of consumption of spirits (*p* < 0.0001; R = 0.4443) in the 30 days prior to the study and “coloured” alcoholic beverages (*p* < 0.0001; R = 3525) in the 30 days prior to the study, while a negative correlation was observed for the frequency of consumption of beer in the 30 days prior to the study (*p* = 0.0002; R = −0.1681). Statistical analysis showed (Table S7), that a significantly higher percentage of respondents with a risky way of drinking alcohol, compared to those with a low level of alcohol consumption, declared drinking beer (66.9% vs. 35.6%, *p* < 0.0001), vodka (50.6% vs. 13.4%, *p* < 0.0001) and coloured alcoholic beverages (29.4% vs. 9.7%, *p* < 0.0001) more often than once a month. Furthermore, it was shown (Table S8) that a significantly higher percentage of respondents with a low risk level of drinking, compared to respondents with a risky drinking style, declared that they had not drunk beer, vodka or coloured beverages in the last month (34.1% vs. 11.3%, *p* < 0.0001, 59.1% vs. 18.8%, *p* < 0.0001, 66.3% vs. 38.7%, *p* < 0.0001, respectively). Additional analyses assessing the influence of the period of data collection on alcohol consumption included in the Supplementary Materials (Tables S15 and S16) showed a relationship only for beer consumption. Previously recruited participants had consumed beer significantly more often in the last 30 days and 12 months prior to the study compared to respondents recruited in the second period of data collection (30.5% vs. 15.6%, *p* = 0.0002 and 49.6% vs. 44.5%, *p* = 0.0005, respectively).

The analysis of the correlation between the average amount of alcoholic beverages consumed on one occasion and the results of the AUDIT test and the knowledge test is presented in Table 7. It was observed that the number of alcoholic beverages consumed on one occasion was not related to the results of the knowledge test (*p* > 0.05). Positive correlations were observed for the AUDIT test results for the total amount of alcoholic beverages consumed on one occasion (*p* < 0.0001; R = 0.4497), beer (*p* < 0.0001; R = 0.2520), wine (*p* = 0.0326; R = 0.0976), spirits (*p* = 0.0431; R = 0.0924) and “coloured” alcoholic beverages (*p* < 0.0001; R = 0.3760). The analysis of the amount of alcoholic beverage consumed on one occasion declared by the respondents showed (Table S9) that more than half drink 1–2 bottles or cans of beer; however, it was shown that a significantly higher percentage of respondents with a risky drinking style compared to those with a low risk level of drinking alcohol declared drinking 3–4 bottles or cans of beer on one occasion (29.4% vs. 9.7%, *p* < 0.0001). In addition, more than half of the respondents declared that they usually drink 1–3 glasses of wine on one occasion, but it was shown that a significantly higher percentage of respondents with a risky drinking style compared to those with a low risk level of drinking alcohol reported drinking more than a bottle of wine on one occasion (13.1% vs. 3.1%, *p* = 0.0007). Statistical analysis also showed that significantly more respondents from the group with a high risk level of alcohol consumption declared drinking more than five glasses of vodka (61.3% vs. 18.8%, *p* < 0.0001) and more than three glasses of coloured alcoholic beverages (28.1% vs. 8.4%, *p* < 0.0001) on one occasion, compared to people with a low risk level of alcohol consumption.

### 3.3. Reasons for Consuming Alcoholic Beverages, Level and Sources of Knowledge about Ethyl Alcohol in the Study Group

Table 8 presents the reasons for the consumption of alcoholic beverages by the respondents divided into groups according to the risk level of alcoholic beverage consumption. Statistical analysis showed that significantly more people from the group at a high risk level of excessive alcohol consumption compared to people from the group at a low risk level of alcohol consumption declared that they drank alcohol because of drinking by friends (43.8% vs. 32.5%, *p* = 0.0157), because of the desire to forget about troubles (25.6% vs. 8.1%, *p* < 0.0001), to improve the mood (70.0% vs. 54.4%, *p* = 0.001), to be courageous (21.3% vs. 7.5%, *p* < 0.0001) or to kill boredom (30.0% vs. 8.1%, *p* < 0.0001). Additional analyses assessing the influence of the period of data collection on the reasons for alcohol consumption included in the Supplementary Materials (Table S18) showed a relationship between the earlier recruitment period and the greater impact of taste on the desire to consume alcoholic beverages (59.6% vs. 42.2%, *p* = 0.0005) and between the later recruitment period and the greater impact of drinking by friends on the desire to consume alcoholic beverages (39.2% vs. 29.1%, *p* = 0.0350).

Table 9 presents the respondents’ knowledge of the impact of alcoholic beverages on human health. There were no statistically significant differences in the respondents’ knowledge of the impact of alcoholic beverages on human health depending on the risk level of alcohol consumption. The analysis of the results of the knowledge test about the health effects of consuming ethyl alcohol (Table S10) showed that compared to respondents with low-risk alcohol consumption, a significantly lower percentage of participants who consume alcohol in a risky style do not know the term “standard drink” in relation to alcohol (39.4% vs. 47.5%, *p* = 0.0213), indicate beer as a drink that can lead to addiction (85.6% vs. 95.9%, *p* < 0.0001), confirm that regular drinking of small doses of alcohol can lead to addiction (78.1% vs. 88.1%, *p* = 0.0034), exclude treating alcohol as a therapeutic agent (53.8% vs. 75.0%, *p* < 0.0001) and also indicate that beer does not make the heart stronger and does not decrease blood pressure (62.5% vs. 71.9%, *p* = 0.0022). At the same time, a higher percentage of respondents with low-risk alcohol consumption, believe that it takes the same amount of time to burn the consumed alcohol as to drink it (22.5% vs. 15.6%, *p* = 0.0121) and deny that the “hangover” lasts up to 20 h and begins after the body has rid the blood of the alcohol (36.9% vs. 29.7%, *p* = 0.0320). Additional analyses assessing the influence of the period of data collection on the level of knowledge about alcohol consumption are included in the Supplementary Materials (Tables S19 and S20). Significant differences were found between the data collection period and the score obtained in the knowledge test. The respondents recruited later had a significantly lower level of knowledge than the participants completing the questionnaire in the first period of data collection (1.45 vs. 8.6%, *p* = 0.0055).

Table 10 presents the respondents’ sources of knowledge about alcohol. It has been shown that a significantly higher percentage of participants with a high-risk level of excessive alcohol consumption indicated their own observations (81.3% vs. 70.5%, *p* = 0.0083) and their peers (48.8% vs. 36.9%, *p* = 0.0126) as sources of knowledge about alcohol, and a smaller percentage indicated teachers (14.4% vs. 21.9%, *p* = 0.0500) compared to people with a low risk level of alcohol consumption.

## 4. Discussion

Consuming alcoholic beverages by young adults is a significant problem not only of a social nature, resulting in injuries, accidents or aggressive behaviour [23], but also in terms of health, leading to addiction and health consequences, such as liver and kidney diseases, problems with the cardiovascular system or anxiety disorders [62,63,64]. Young adults consume alcoholic beverages during heavy drinking sessions, which are defined as the consumption of 60 g or more of pure ethanol consumed at least once a month, with the frequency of heavy drinking sessions highest in those aged 20–24 and especially high among young men [2,65]. According to current reports, the frequency of consumption of alcoholic beverages peaks at the age of 25–45 [2,66], and the level of knowledge of young adults about the effects of alcohol on health is insufficient [29,30].

Data in the literature indicate an increasing percentage of adolescents and young adults who consume alcoholic beverages, but also declare themselves as binge drinking or drinking alcohol every day [67,68,69]. In this study, the majority of respondents declared drinking alcoholic beverages in the last 12 months, and even in the last 30 days. Moreover, a significant proportion of respondents reported having become inebriated at least several times in their life. Research conducted in the younger age group indicates the problem of regular drinking of alcohol by adolescents. In Australia, more than half of the surveyed adolescents aged 12–17 declared that they had drunk alcohol in the last year [70], while regular drinking was reported by slightly more than 10% of Canadian youths aged 12–17 [71]. Although the results of more recent studies indicate a reduction in the frequency of drinking alcoholic beverages among young adults, high-risk alcohol consumption is still observed among students [72,73]. The authors of the research describe students as heavy and risky drinkers. Davoren et al. [74] showed that the vast majority of the studied students were in the group of risky consumption of ethanol, and about 20% of them declared regular consumption of alcoholic beverages on weekdays. On the other hand, Boniface et al. [75] and John et al. [76] reported that the majority of young adults surveyed reported exceeding consumption limits and binge drinking during the last week. Another study by Black and Monrouxe [77] shows that a significant proportion of students report consuming at least 15 units of alcohol per week. Lee et al. [78] analysed the influence of the type of work on the frequency of consumption of alcoholic beverages, showing that the unemployed were much less likely to develop heavy drinking episodes (HED) than those working full-time. Moreover, the unemployed consumed less alcoholic beverages per week and experienced fewer health consequences, while in the group of full-time workers the consumption of alcoholic beverages was the highest, and they were much more likely to exceed alcohol consumption limits. The possible causes of alcohol abuse by working young adults may include the higher income of full-time workers, workplace amenities such as “*happy hour*” and the desire to reduce work-related stress, but also the need to feel more independent from parents [79,80,81]. 

In this study, the AUDIT test, which is a commonly used tool, was used to assess the drinking style of alcoholic beverages in a group of young adults. It was shown that the majority of the studied people were at a low risk level of alcoholic beverage consumption, with a greater percentage being women. In other studies, which also used the AUDIT test, an increase in the percentage of respondents drinking in a risky manner was observed in the group of students. The study by Beenstock [82] showed an increase in the percentage of people who obtained at least 8 points in the AUDIT test compared to previous years [83] (80% vs. 65%), which indicates an increase in the percentage of students abusing alcohol. In this study, a third of the respondents obtained at least 8 points in the AUDIT test. On the other hand, Messina et al. [84] conducted a study in a group of almost 2000 students using the AUDIT test, and the results show that more than half of the respondents had a high risk level of ethanol consumption, with a greater percentage of them being women. In a study by Heather [73], which analysed risky drinking among students at seven UK universities, the author did not find a significant difference in the AUDIT scores for men and women. Similar results were obtained in the study by O’Brien [85], which showed that students belonging to a sports team sponsored by the alcohol industry displayed more risky drinking behaviour, regardless of gender. Taking into account the research indicating an indirect relationship between alcohol sponsorship in sports and the subsequent development of risky behaviours related to alcohol consumption [86], the need to prohibit the advertising of alcoholic beverages and sports sponsorship by the alcohol industry is indicated [87,88].

The results of this study indicate that the consumption of alcoholic beverages for the first time by respondents who drink in a hazardous manner occurred around the age of 10–15 years old. The problem of an earlier alcohol initiation is indicated by the authors of many studies conducted in Poland [89,90,91] and around the world [5,92,93,94]. According to Grant and Dawson [95], starting alcohol consumption earlier (before the age of 15 years old) is associated with an increased risk of developing alcohol-related problems later in life. Guttmmannova et al. [96] attempted to determine the effect of the age of starting alcohol use on alcohol abuse and alcohol dependence in adulthood. It was assumed that the period of early adolescence (11–14 years) is a sensitive period of development, in which starting to drink alcohol is particularly harmful. However, it has been shown that no period of puberty is more sensitive than others. It has been observed that people who started drinking alcohol regularly before the age of 21 years old had a higher rate of alcohol dependence in adulthood, while starting regular alcohol use at the age of 14 years old or earlier was not associated with a higher risk of alcohol dependence than starting regular alcohol use in middle or late puberty. The age of alcohol initiation may be influenced by many factors, such as socio-demographic variables, family, peers, personality traits and behavioural variables [97,98]. Alcohol consumption between the ages of 15–17 and earlier may be associated with damage to the developing brain [99], lead to the development of alcohol dependence later [100] and also increase the risk of disability [101]. This underscores the importance of screening for alcoholic beverage consumption among children and adolescents in order to implement appropriate measures to delay the age of first exposure to ethyl alcohol [102,103].

This study reported that young people with a high-risk level of alcohol consumption were more likely to consume alcoholic beverages at a social gathering for the first time. Similar results were obtained in other studies which showed that the main places where adolescents first consumed alcohol were at parties with friends or family or at street events [104,105]. Among the factors influencing the age of alcohol initiation there are social factors such as the company of peers or the family situation [97,98]. In addition, Osaki et al. [106] indicate that the first interaction of young people with alcohol is the result of the behaviour of family members and peers, with the influence of peers being much stronger. The minimum age limits for consumption of alcoholic beverages are less applicable in social settings such as the family home and social gatherings. Young people can easily succumb to the incentives to consume alcoholic beverages as part of their daily interactions with peers. Research has shown that peer drinking influences alcohol consumption among friends, while widespread disapproval of drinking among peers is associated with delayed initiation and lower alcohol consumption [65]. The results of the analysed studies indicate that the social space plays an important role in the first experiences related to alcohol consumption by adolescents. Therefore, it seems very important to plan and undertake appropriate educational activities with increasing involvement of parents, aimed at creating safe social spaces for young people and preventing the development of alcohol-related disorders in young people [107,108]. 

The choice and preferences of alcoholic beverages are related to many factors, including risky one-time alcohol use [109], daily alcohol consumption [109,110], health status, age and gender [111,112,113,114]. The analysis of the drinking style of alcoholic beverages by the respondents of this study showed that they most willingly drink beer, then wine and least frequently vodka. Other Polish questionnaire studies also showed that young adults most often drink beer [115,116]; a similar style of alcoholic beverage consumption is also observed in adolescents [117,118,119]. It should be emphasized that the current study also showed that significantly more people with a risky drinking style, compared to people with a low-risk level of alcohol consumption, drank vodka, which was also the first alcoholic drink they consumed. The available literature does not provide data on the preferences of alcoholic beverages in young adults who drink in a risky manner. In the study conducted by Cook et al. [120] in the group of adult men, no correlation was found between the amount of ethanol consumed from beer, wine and spirits and the results of the AUDIT test. It seems that this issue should be addressed in well-planned scientific studies, because our results show that risky drinkers of alcoholic beverages started their alcohol consumption with spirits and continue to drink them later in life, which may contribute to the faster development of health problems [121,122,123]. 

Studies show a significant association between the COVID-19 pandemic and the increase in alcoholic beverage consumption, exceeding recommended consumption limits and binge drinking [12,124,125]. According to psychological behavioural theories, social and environmental constraints related to the COVID-19 pandemic may have resulted in an increase in alcohol consumption to deal with the negative effects of lock-down stress [126]. In this study, it was observed that the consumption of alcoholic beverages among the respondents did not change. A significant proportion of respondents reported a reduction in consumption, but a significantly higher percentage of risky drinkers reported an increase in alcohol consumption during the pandemic. In studies by other authors that analysed changes in the frequency of alcohol consumption during the COVID-19 pandemic compared with behaviour before the pandemic, both increased and decreased alcohol consumption in various population groups were reported [127]. Moreover, many studies have observed elevated AUDIT scores compared to the pre-pandemic period [128,129,130,131], and some studies have also found an increase in the frequency of drinking before 5.00 p.m. [132]. On the other hand, Killgore et al. [130] showed a positive correlation between the length of isolation and the average result obtained in the AUDIT test. Although a study by Sallie et al. [133] found that overall AUDIT scores decreased during the pandemic, respondents who increased their alcohol consumption during the pandemic also increased their drinking frequency. In addition, Weerakoon et al. [134] found that pre-pandemic alcohol abusers were more likely to increase their alcohol consumption during the pandemic compared to those who did not drink at a risky level. According to Calina et al. [135], in the context of the COVID-19 pandemic, alcohol consumption is a way for many people to relieve stress. The COVID-19 pandemic can affect the amount of alcohol consumed and the way of someone relates to that consumption, but it can also cause medium to long-term changes in health behaviour and the health status of many people. Due to the high global consumption of alcohol and the consequences related to its consumption, such as, for example, the weakening of the immune system [136], or the impaired development of immunity in response to vaccination [137], OECD [18] points out that comprehensive measures, including limiting the promotion of alcoholic beverages, or changes in the availability of alcoholic beverages, may be effective and economically beneficial in the fight against excessive drinking during a pandemic. 

The present study showed that the majority of respondents consumed alcoholic beverages to improve their mood and feel better. Similarly, other authors indicate that young people most often drink alcoholic beverages in order to improve their mood, escape from problems or become like their peers [138,139,140]. A study of Italian students [84] showed that a significant proportion of high-risk respondents consume alcoholic beverages to cope with negative emotions, and low-risk individuals consume them under the influence of their peers. Similar results were obtained in our study, in which a significant proportion of the respondents, especially those from the group with a high-risk level of excessive alcohol consumption, also indicated that ethyl alcohol is helpful in overcoming difficulties such as trouble, lack of courage or overcoming boredom, while people from the low-risk group declared that they consume alcoholic beverages in order to improve mood and under the influence of friends. During a pandemic, an additional cause of increased alcohol consumption is increased stress, greater availability of alcohol and boredom, moreover, the stress associated with a pandemic increases the amount of alcohol drunk, which poses a health risk both from the perspective of the individual and wider society [12]. These results confirm the necessity of conducting educational activities aimed at increasing consumer awareness of the risks and negative effects of excessive alcohol consumption in order to cope with difficult emotions. 

There are few studies assessing the level of knowledge of young people about the health effects of alcohol. The available literature which analysed respondents’ knowledge of alcohol focused on specific aspects, such as the energy value of alcoholic beverages [141], knowledge of the term “standard drink” [142,143,144] or their impact on health [30,145] and foetal development [146]. However, there are no studies that assess all these aspects in a single questionnaire. For this reason, this study includes questions that test the level of knowledge of all of the abovementioned aspects. The results showed that the respondents’ level of knowledge was average or low. Respondents with a high-risk level of alcohol consumption more often showed a lack of knowledge of the term “standard drink” in relation to alcoholic beverages. In the study by Vallance et al. [144], less than one third of the respondents correctly indicated the number of standard drinks in one package of various types of alcoholic beverages. Moreover, the older participants, with greater health awareness, more often gave correct answers compared to the younger and less health-conscious participants. In the study by Chodkiewicz [40], students’ beliefs about popular stereotypes about ethanol were tested. It was shown that almost 25% of participants did not associate the regular consumption of beer with the possibility of alcohol dependence, and over 80% of respondents denied the possibility of addiction resulting from regular binge drinking. Almost half of the respondents indicated ethyl alcohol as a treatment adjuvant. In the present study, it was observed that people with a high-risk level of excessive alcohol consumption responded more frequently, similar to the participants in the Chodkiewicz study [40]. In a study by Messina et al. [84], a relationship was observed between risky alcohol consumption, determined on the basis of the AUDIT test results, and better knowledge of the respondents. Based on these results, the authors concluded that the mere awareness of the effects of alcohol on the body is not sufficient to prevent the occurrence of disorders associated with its excessive consumption. The authors emphasise the need for further research, as well as intensified educational activities, due to the long-term health consequences of alcohol abuse at a young age, as well as the high social and financial costs of ethanol addiction [147,148]. 

## 5. Limitation 

The questionnaire on the knowledge about the health impact of ethyl alcohol used in the study was developed on the basis of questionnaires by other authors. The created questionnaire contained three questions to which there are no unambiguous answers in the currently available scientific literature, and which are formulated in an ambiguous manner, which could raise doubts in the respondents and affect the answers provided. Therefore, for the purposes of future research, better tools should be developed to assess the level of knowledge about the health effects of ethanol. An additional limitation of the study was that it was conducted in two stages (from June 2021 to September 2021 and from March 2022 to April 2022), which could have had a direct impact on the results of this study due to the possible differences in alcohol consumption during different periods of the year by study participants. In order to reduce the impact of this limitation, additional statistical analyses were performed and discussed in the results. Another limitation of the study is the fact that there is little possibility of generalising the results, because the sample is not fully representative.

## 6. Conclusions

The results of the presented study show that young adults, especially men, consume alcoholic beverages in a risky manner, and the main motive for reaching for alcoholic beverages is the desire to improve mood. Additionally, knowledge about the influence of ethanol on health among young adults is insufficient, and the most frequently mentioned sources of obtaining knowledge are the Internet and participants’ own observations, which confirms the research hypothesis made at the beginning of the study. Based on the presented results, it can be concluded that there is a need for more research, especially among young adults, into controlling alcohol abuse, as well as regular educational activities increasing the level of reliable knowledge about the impact of ethyl alcohol on the body. Educational and preventive activities should be based on the promotion of limiting the consumption of alcoholic beverages in social media, as well as using the latest technologies based on attributes such as anonymity and self-sufficiency, which are important for young adults. In addition, it is wise to take all necessary measures to prevent alcohol abuse problems from escalating during the coronavirus pandemic. Therefore, it seems warranted for governmental authorities to monitor alcohol consumption on a regular basis to determine the long-term health effects of drinking during a pandemic. 

## Figures and Tables

**Table 1:** Knowledge Index Score Interpretation.

Number of Points	Interpretation
28–21	High level of knowledge
20–14	Average level of knowledge
<14	Low level of knowledge

**Table 2:** Anthropometric characteristics of the studied group of participants by gender.

Parameter		Total (*n* = 480)	Females (*n* = 271)	Males (*n* = 209)	*p* ^2^
Age (years)	Mean ± SD	24.5 ± 4.0	23.5 ± 3.1	25.9 ± 4.6	<0.0001
	Median ^1^ (min–max)	24 (18–35)	23 (18–35)	25 (18–35)	
Weight (kg)	Mean ± SD	70.4 ± 16.7	61.8 ± 12.1	81.5 ± 15.3	<0.0001
	Median (min–max)	68 (42–132)	59 (42–112)	80 (46–132)	
Height (cm)	Mean ± SD	173.1 ± 9.9	166.8 ± 6.1	181.4 ± 7.5	<0.0001
	Median (min–max)	171 (145–203)	167 (145–185)	180 (160–203)	
BMI (kg/m^2^)	Mean ± SD	23.3 ± 4.2	22.2 ± 3.9	24.7 ± 4.3	<0.0001
	Median (min–max)	22.6 (16.1–41.0)	21.5 (16.5–40.6)	24.3 (16.1–41.0)	

^1^ non-parametric distribution (Kolmogorov–Smirnov test with Lillefors correction; *p* < 0.05); ^2^ comparison using Pearson’s chi-square test (χ^2^); *p* < 0.05.

**Table 3:** Socio-demographic characteristics of the studied group of participants by gender.

Parameter	Total (*n* = 480)	Females (*n* = 271)	Males (*n* = 209)	*p*
Marital status				
Single	428 (89.2%)	249 (91.9%)	179 (85.7%)	0.0030
Married	44 (9.2%)	22 (8.1%)	22 (10.5%)	
Divorced	8 (1.7%)	0 (0.0%)	8 (3.8%)	
Widowed	0 (0.0%)	0 (0.0%)	0 (0.0%)	
Size of the place of residence				
Village	76 (15.8%)	49 (18.1%)	27 (12.9%)	0.0055
City up to 5000 residents	24 (5.0%)	5 (1.9%)	19 (9.1%)	
City between 5000 and 50,000 residents	70 (14.6%)	41 (15.1%)	29 (13.9%)	
City between 50,000 and 200,000 residents	49 (10.2%)	29 (10.7%)	20 (9.6%)	
City over 200,000 residents	261 (54.4%)	147 (54.2%)	114 (54.6%)	
Profession				
Student	270 (56.3%)	194 (71.6%)	76 (36.4%)	<0.0001
Full-time job	171 (35.6%)	65 (24.0%)	20 (9.6%)	
Odd job	28 (5.8%)	8 (2.9%)	106 (50.7%)	
Unemployed	11 (2.3%)	4 (1.5%)	7 (3.4%)	

Values express counts (*n*) and percentages (%). Statistically significant differences between the sexes were analysed using Pearson’s chi-square test (χ^2^).

**Table 4:** Drinking risk groups based on the AUDIT test results by gender.

Interpretation	Total (*n* = 480)	Females (*n* = 271)	Males (*n* = 209)	*p*
Low-risk alcohol consumption High-risk alcohol consumption	320 (66.7%) 160 (33.3%)	205 (75.7%) 66 (24.4%)	115 (55.0%) 94 (45.0%)	<0.0001

Values express counts (*n*) and percentages (%). Statistically significant differences between the sexes were analysed using Pearson’s chi-square test (χ^2^).

**Table 5:** The analysis of the correlation between age and place of alcohol initiation, the type of alcoholic beverages consumed first, the type of alcoholic beverages consumed most often, changes in the consumption of alcoholic beverages under the COVID-19 pandemic, the amount of money spent on the purchase of alcoholic beverages and the frequency of binge drinking, and the results of the AUDIT test and the knowledge test in the group of young adults who participated in the study.

Parameter	AUDIT Test		Knowledge Test	
	*p*	R ^1^	*p*	R ^1^
Age of alcohol initiation	0.0001	−0.1730	0.0920	−0.0770
Place of alcohol initiation	0.1412	0.0673	0.0190	−0.1071
The type of alcoholic drink consumed first	0.0327	−0.0975	0.8585	0.0082
The type of alcoholic beverages consumed most often currently	<0.0001	0.1830	0.2762	−0.0498
The impact of the COVID-19 pandemic on the frequency of consumption of alcoholic beverages	0.1228	−0.0705	0.2517	0.0524
The amount of money spent on purchasing alcoholic beverages	<0.0001	0.2395	0.0803	−0.0114
The frequency of binge drinking	<0.0001	0.3485	0.7492	0.0146

^1^ non-parametric Spearman test.

**Table 6:** The analysis of the correlation between the frequency of consumption of various types of alcoholic beverages and the results of the AUDIT test and the knowledge test in the group of young adults who participated in the study.

Parameter	AUDIT Test		Knowledge Test	
	*p*	R ^1^	*p*	R ^1^
The frequency of consumption of alcoholic beverages	0.2602	0.0515	0.0623	−0.0852
The frequency of consumption of beer in the 12 months prior to the test	0.2536	−0.0522	0.4605	−0.0338
The frequency of consumption of wine in the 12 months prior to the test	0.2304	0.0548	0.9000	0.0058
The frequency of consumption of spirits in the 12 months prior to the study	0.7130	0.0168	0.1486	0.0660
The frequency of consumption of “coloured” alcoholic beverages in the 12 months prior to the study	<0.0001	0.4153	0.3970	0.0388
The frequency of consumption of beer in the 30 days prior to the test	0.0002	−0.1681	0.5787	−0.0254
The frequency of consumption of wine in the 30 days prior to the test	0.4053	0.0381	0.3857	0.0397
The frequency of consumption of spirits in the 30 days prior to the test	<0.0001	0.4443	0.9570	−0.0025
The frequency of consumption of “coloured” alcoholic beverages in the 30 days prior to the test	<0.0001	0.3525	0.7940	−0.0120

^1^ non-parametric Spearman test.

**Table 7:** The analysis of the correlation between the average amount of alcoholic beverages consumed on one occasion and the results of the AUDIT test and the knowledge test in the group of young adults who participated in the study.

Parameter	AUDIT Test		Knowledge Test	
	*p*	R ^1^	*p*	R ^1^
Amount of alcoholic beverages consumed on one occasion	<0.0001	0.4497	0.4380	−0.0355
Amount of beer consumed on one occasion	<0.0001	0.2520	0.4276	−0.0363
Amount of wine consumed on one occasion	0.0326	0.0976	0.2184	−0.0562
Amount of spirits consumed on one occasion	0.0431	0.0924	0.1459	0.0665
Amount of “coloured” alcoholic beverages consumed on one occasion	<0.0001	0.3760	0.1844	0.0607

^1^ non-parametric Spearman test.

**Table 8:** Reasons for consuming alcoholic beverages in the study group divided according to the risk level of alcohol consumption.

Parameter	Total (*n* = 480)	Low Level of Risk (*n* = 320)	Risky Consumption of Ethyl Alcohol (*n* = 160)	*p*
Why do you drink alcoholic beverages?				
Because friends drink	174 (36.2%)	104 (32.5%)	70 (43.8%)	0.0157
To forget about troubles	67 (13.9%)	26 (8.1%)	41 (25.6%)	<0.0001
To have fun, be in a better mood	286 (59.6%)	174 (54.4%)	112 (70.0%)	0.0010
To take courage	58 (12.1%)	24 (7.5%)	34 (21.3%)	<0.0001
To kill boredom	74 (15.4%)	26 (8.1%)	48 (30.0%)	<0.0001
It is tasty	227 (47.3%)	149 (46.6%)	78 (48.8%)	0.6509
Out of curiosity	28 (5.8%)	21 (6.6%)	7 (4.4%)	0.3351
Other				
- Type of work	2 (0.4%)	1 (0.3%)	1 (0.6%)	0.6163
- Support sleep	1 (0.2%)	1 (0.3%)	0 (0.0%)	0.4790
- I don’t drink alcohol	9 (1.9%)	9 (2.8%)	0 (0.0%)	0.0322

Values express counts (*n*) and percentages (%). Statistically significant differences between the risk level of alcohol consumption were analysed using Pearson’s chi-square (χ^2^).

**Table 9:** The level of knowledge about ethyl alcohol in the study group divided according to the risk level of alcohol consumption.

Interpretation	Total (*n* = 480)	Low Level of Risk (*n* = 320)	Risky Consumption of Ethyl Alcohol (*n* = 160)	*p*
High knowledge index	42 (8.8%)	28 (8.8%)	14 (8.8%)	0.4432
Average knowledge index	285 (59.4%)	184 (57.5%)	101 (63.1%)	
Low knowledge index	153 (31.8%)	108 (33.8%)	45 (28.1%)	

Values express counts (*n*) and percentages (%). Statistically significant differences between the risk levels of alcohol consumption were analysed using Pearson’s chi-square test (χ^2^).

**Table 10:** Sources of knowledge on ethyl alcohol in the study group divided according to the risk level of alcohol consumption.

Parameter	Total (*n* = 480)	Low Level of Risk (*n* = 320)	Risky Consumption of Ethyl Alcohol (*n* = 160)	*p*
Where do you get information on the impact of alcohol on human health from?				
Internet	384 (80.0%)	259 (80.9%)	125 (78.1%)	0.4677
Television	75 (15.6%)	51 (15.9%)	24 (15.0%)	0.7897
Radio	22 (4.6%)	15 (4.7%)	7 (4.4%)	0.8774
Own observation	354 (73.7%)	224 (70.0%)	130 (81.3%)	0.0083
Press	37 (7.7%)	24 (7.5%)	13 (8.1%)	0.8088
Teachers	93 (19.4%)	70 (21.9%)	23 (14.4%)	0.0500
Parents	99 (20.6%)	62 (19.4%)	37 (23.1%)	0.3385
Peers	196 (40.8%)	118 (36.9%)	78 (48.8%)	0.0126
Books	71 (14.8%)	50 (15.6%)	21 (13.1%)	0.4671
Healthcare workers	78 (16.3%)	53 (16.6%)	25 (15.6%)	0.7930
Other				
- Social campaigns	1 (0.2%)	1 (0.3%)	0 (0.0%)	0.4790
- I’m not interested	1 (0.2%)	1 (0.3%)	0 (0.0%)	0.4790

Values express counts (*n*) and percentages (%). Statistically significant differences between the risk level of alcohol consumption were analysed using Pearson’s chi-square test (χ^2^).

## Data Availability

Not applicable.

## Supplementary Materials

The following supporting information can be downloaded at: https://www.mdpi.com/article/10.3390/ijerph192315425/s1.

## References

[1] ESPAD Report 2019: Results from European School Survey Project on Alcohol and Other Drugs. http://www.espad.org/sites/espad.org/files/2020.3878_EN_04.pdf.

[2] World Health Organization Global Status Report on Alcohol and Health. https://www.who.int/publications/i/item/9789241565639.

[3] Chaiyasong S., Huckle T., Mackintosh A.M., Meier P., Parry C.D., Callinan S., Cuong P.V., Kazantseva E., Gray-Phillip G., Parker K. (2018). Drinking patterns vary by gender, age and country-level income: Cross-country analysis of the International Alcohol Control Study. Drug Alcohol Rev..

[4] Soundararajan S., Narayanan G., Agrawal A., Prabhakaran D., Murthy P. (2017). Relation between age at first alcohol drink & adult life drinking patterns in alcohol-dependent patients. Indian J. Med. Res..

[5] Ahuja M., Awasthi M., Records K., Lamichhane R.R. (2021). Early Age of Alcohol Initiation and its Association with Suicidal Behaviors. Subst. Use Misuse.

[6] Veerbeek M.A., Ten Have M., van Dorsselaer S.A., Voshaar R.C.O., Rhebergen D., Willemse B.M. (2019). Differences in alcohol use between younger and older people: Results from a general population study. Drug Alcohol Depend..

[7] Visontay R., Mewton L., Sunderland M., Prior K., Slade T. (2020). Changes over time in young adults’ harmful alcohol consumption: A cross-temporal meta-analysis using the AUDIT. Drug Alcohol Depend..

[8] Burgess A., Yeomans H., Fenton L. (2022). ‘More options…less time’ in the ‘hustle culture’ of ‘generation sensible’: Individualization and drinking decline among twenty-first century young adults. Br. J. Sociol..

[9] Torronen J., Roumeliotis F., Samuelsson E., Kraus L., Room R. (2019). Why are young people drinking less than earlier? Identifying and specifying social mechanisms with a pragmatist approach. Int. J. Drug Policy.

[10] Irizar P., Puddephatt J.A., Warren J.G., Field M., Jones A., Rose A.K., Gage S.H., Goodwin L. (2022). “Drinkers Like Me”: A Thematic Analysis of Comments Responding to an Online Article about Moderating Alcohol Consumption. Front. Psychol..

[11] Organisation for Economic Co-Operation and Development Preventing Harmful Alcohol Use. https://www.oecd-ilibrary.org/sites/6e4b4ffb-en/index.html?itemId=/content/publication/6e4b4ffb-en.

[12] Grossman E.R., Benjamin-Neelon S.E., Sonnenschein S. (2020). Alcohol Consumption during the COVID-19 Pandemic: A Cross-Sectional Survey of US Adults. Int. J. Environ. Res. Public Health.

[13] Pollard M.S., Tucker J.S., Green H.D. (2020). Changes in Adult Alcohol Use and Consequences during the COVID-19 Pandemic in the US. JAMA Netw. Open.

[14] Chodkiewicz J., Talarowska M., Miniszewska J., Nawrocka N., Bilinski P. (2020). Alcohol Consumption Reported during the COVID-19 Pandemic: The Initial Stage. Int. J. Environ. Res. Public Health.

[15] Steffen J., Schlichtiger J., Huber B.C., Brunner S. (2021). Altered alcohol consumption during COVID-19 pandemic lockdown. Nutr. J..

[16] COVID-19 and Increased Alcohol Consumption: NANOS Poll Summary Report. https://www.ccsa.ca/covid-19-and-increased-alcohol-consumption-nanos-poll-summary-report.

[17] Coakley K.E., Lardier D.T., Holladay K.R., Amorim F.T., Mechler H., Zuhl M.N. (2021). Mental Health Severity Is Associated with Increases in Alcohol Consumption in Young Adult Students during the COVID-19 Pandemic. Alcohol. Treat. Q..

[18] Patrick M.E., Terry-McElrath Y.M., Miech R.A., Keyes K.M., Jager J., Schulenberg J.E. (2022). Alcohol use and the COVID-19 pandemic: Historical trends in drinking, contexts, and reasons for use among U.S. adults. Soc. Sci. Med..

[19] Dubey S., Biswas P., Ghosh R., Chatterjee S., Dubey M.J., Chatterjee S., Lahiri D., Lavie C.J. (2020). Psychosocial impact of COVID-19. Diabetes Metab. Syndr..

[20] The Effect of COVID-19 on Alcohol Consumption, and Policy Responses to Prevent Harmful Alcohol Consumption. https://read.oecd-ilibrary.org/view/?ref=1094_1094512-803wufqnoe&title=The-effect-of-COVID-19-on-alcohol-consumption-and-policy-responses-to-prevent-harmful-alcohol-consumption.

[21] Boumparis N., Schulte M.H., Kleiboer A., Huizink A., Riper H. (2021). A mobile intervention to promote low-risk drinking habits in young adults: Protocol for a randomized controlled trial. JMIR Res. Protoc..

[22] World Health Organization Alcohol Does Not Protect against COVID-19; Access Should Be Restricted during Lockdown. https://www.euro.who.int/en/health-topics/disease-prevention/alcohol-use/news/news/2020/04/alcohol-does-not-protect-against-covid-19-access-should-be-restricted-during-lockdown.

[23] Iranpour A., Nakhaee N. (2019). A Review of Alcohol-Related Harms: A Recent Update. Addict. Health.

[24] Pompili M., Serafini G., Innamorati M., Dominici G., Ferracuti S., Kotzalidis G.D., Serra G., Girardi P., Janiri L., Tatarelli R. (2010). Suicidal behaviour and alcohol abuse. Int. J. Environ. Res. Public Health.

[25] World Health Organization Social Determinants of Health and Well-Being among Young People. Health Behaviour in School-aged Children (HBSC) Study: International Report from the 2009/2010 Survey. https://www.euro.who.int/data/assets/pdf_file/0007/167281/E96444_part1.pdf.

[26] Hibell B., Guttormsson U. A Supplement to the 2011 ESPAD Report. https://www.can.se/app/uploads/2019/12/full-report-supplement-to-the-2011-espad-report.pdf.

[27] Doyle A. Alcohol Consumption, Alcohol-Related Harm, and Alcohol Policy in Ireland. https://www.drugsandalcohol.ie/34737/1/Drugnet_Ireland_78.pdf.

[28] De Visser R.O., Birch J.D. (2012). My cup runneth over: Young people’s lack of knowledge of low-risk drinking guidelines. Drug Alcohol Rev..

[29] Isted A., Fiorini F., Tillmann T. (2015). Knowledge gaps and acceptability of abbreviated alcohol screening in general practice: A cross-sectional survey of hazardous and non-hazardous drinkers. BMC Fam. Pract..

[30] Alves R.F., Precioso J., Becoña E. (2021). Alcohol-related knowledge and attitudes as predictors of drinking behaviours among Portuguese university students. Alcohol. Drug Addict..

[31] Zarzeczna-Baran M., Bandurska E., Nowalińska M., Zawiślińska L. (2016). Wiedza studentów gdańskich uczelni wyższych na temat skutków spożywania alkoholu przez kobiety w ciąży (eng. Knowledge of Gdańsk universities students on the effects of alcohol consumption by pregnant women). Ann. Acad. Med. Gedan.

[32] Karakuła H. (2009). Style spożywania alkoholu przez studentów lubelskich uczelni wyższych oraz ich opinie dotyczące wybranych aspektów problematyki alkoholowej (eng. The styles of alcohol consumption by students of Lublin universities and their opinions on selected aspects of alcohol issues). Ann. Fam. Sci..

[33] Sidor K., Makara-Studzińska M. (2012). Profil studentów pijących ryzykownie w grupie studentów Uniwersytetu Medycznego w Lublinie (eng. Profile of high-risk drinkers in the group of students of the Medical University of Lublin). Hygeia Public Health.

[34] Klimkiewicz A., Jakubczyk A., Mach A., Abramowska M., Szczypiński J., Berent D., Skrzeszewski J., Witkowski G., Wojnar M. (2021). Psychometric properties of the polish version of the Alcohol Use Disorders Identification Test (AUDIT). Drug Alcohol Depend..

[35] Naderifar M., Goli H., Ghaljaie F. (2017). Snowball sampling: A purposeful method of sampling in qualitative research. Strides Dev. Med. Educ..

[36] Stelmach-Mardas M., Kleiser C., Uzhova I., Peñalvo J.L., La Torre G., Palys W., Lojko D., Nimptsch K., Suwalska A., Linseisen J. (2016). Seasonality of food groups and total energy intake: A systematic review and meta-analysis. Eur. J. Clin. Nutr..

[37] Guelinckx I., Devlieger R., Vansant G. (2011). Alcohol during pregnancy and lactation: Recommendations versus real intake. Arch. Public Health.

[38] Emonts P., Capelle X., Grandfils S., Petit P., Bücheler V., Rigo V. (2019). Alcool, grossesse et al.laitement [Alcohol, pregnancy and breast-feeding]. Rev. Med. Liege.

[39] Australian Government, Department of Health and Aged Care Alcohol during Pregnancy and Breastfeeding. https://www.health.gov.au/health-topics/alcohol/alcohol-throughout-life/alcohol-during-pregnancy-and-breastfeeding.

[40] Chodkiewicz J. (2006). Picie alkoholu oraz wiedza o jego działaniu wśród studentów łódzkich szkół wyższych (eng. Drinking alcohol and knowledge about its effects among students of Lodz universities). Alcohol. Drug Addict..

[41] Sierosławski J. Raport z Badania Ankietowego na Temat Używania Substancji Psychoaktywnych Przez Studentów (Eng. Report from a Survey on the Use of Psychoactive Substances by Polish Students). https://www.narkomania.org.pl/czytelnia/studenci-2004-raport-z-badania-ankietowego-na-temat-uzywania-substancji-psychoaktywnych-przez-studentow/.

[42] Pretendent Diagnoza Problemów Dotyczących Zjawiska Spożywania Alkoholu, Zażywania Narkotyków, Nikotynizmu Oraz Występowania Przemocy Wśród Dzieci i Młodzieży w Radomsku (Eng. Diagnosis of Problems Related to Alcohol Consumption, Drug Use, Nicotinism and the Occurrence of Violence among Children and Adolescents in Radomsko). https://bip.radomsko.pl/res/serwisy/bip-umradomsko/komunikaty/_008_016_229543.pdf.

[43] Autodiagnoza Jak Ocenić Swoje Picie? (Eng. Self-Diagnosis. How Do I Rate My Drinking?). https://www.parpa.pl/images/file/Autodiagnoza_1.pdf.

[44] Żołnierczuk-Kieliszek D., Kulik T.B., Sidor R., Janiszewska M., Stefanowicz A., Pacian A., Pacian J. (2013). Zachowania zdrowotne związane ze spożyciem alkoholu i wiedza młodzieży gimnazjalnej na temat skutków nadużywania alkoholu. (eng. Health behaviors related to alcohol consumption and knowledge of junior high school students about the effects of alcohol abuse). Gen. Med. Health Sci..

[45] Jaworowski S., Walter G., Soh N., Dror Y.F., Mergui J., Gropp C., Haber P.S. (2014). A Validated Questionnaire to Assess the Knowledge of Psychiatric Aspects of Alcohol Use Disorder. Subst. Abus..

[46] Ipsos MRBI (2012). Alcohol: Public Knowledge, Attitudes and Behaviour.

[47] Abrahao K.P., Salinas A.G., Lovinger D.M. (2017). Alcohol and the Brain: Neuronal Molecular Targets, Synapses, and Circuits. Neuron.

[48] Mackus M., Loo A., Garssen J., Kraneveld A.D., Scholey A., Verster J.C. (2020). The Role of Alcohol Metabolism in the Pathology of Alcohol Hangover. J. Clin. Med..

[49] McNabb S., Harrison T.A., Albanes D., Berndt S.I., Brenner H., Caan B.J., Campbell P.T., Cao Y., Chang-Claude J., Chan A. (2020). Meta-analysis of 16 studies of the association of alcohol with colorectal cancer. Int. J. Cancer.

[50] Park S.Y., Wilkens L.R., Setiawan V.W., Monroe K.R., Haiman C.A., Le Marchand L. (2019). Alcohol Intake and Colorectal Cancer Risk in the Multiethnic Cohort Study. Am. J. Epidemiol..

[51] Day E., Rudd J. (2019). Alcohol use disorders and the heart. Addiction.

[52] Spaggiari G., Cignarelli A., Sansone A., Baldi M., Santi D. (2020). To beer or not to beer: A meta-analysis of the effects of beer consumption on cardiovascular health. PLoS ONE.

[53] Konjengbam H., Meitei S.Y. (2020). Association of kidney stone disease with dietary factors: A review. Anthropol. Rev..

[54] Weiskirchen S., Weiskirchen R. (2016). Resveratrol: How Much Wine Do You Have to Drink to Stay Healthy?. Adv. Nutr..

[55] Verster J.C., Stephens R., Penning R., Rohsenow D., McGeary J., Levy D., McKinney A., Finnigan F., Piasecki T.M., Adan A. (2010). The alcohol hangover research group consensus statement on best practice in alcohol hangover research. Curr. Drug Abus. Rev..

[56] Palmer E., Tyacke R., Sastre M., Lingford-Hughes A., Nutt D., Ward R.J. (2019). Alcohol Hangover: Underlying Biochemical, Inflammatory and Neurochemical Mechanisms. Alcohol Alcohol..

[57] Snopek L., Mlcek J., Sochorova L., Baron M., Hlavacova I., Jurikova T., Kizek R., Sedlackova E., Sochor J. (2018). Contribution of Red Wine Consumption to Human Health Protection. Molecules.

[58] Srinivasan S., Dubey K.K., Singhal R.S. (2019). Influence of food commodities on hangover based on alcohol dehydrogenase and aldehyde dehydrogenase activities. Curr. Res. Food Sci..

[59] Parast L., Meredith L.S., Stein B.D., Shadel W.G., D’Amico E.J. (2018). Identifying adolescents with alcohol use disorder: Optimal screening using the National Institute on Alcohol Abuse and Alcoholism screening guide. Psychol. Addict. Behav. J. Soc. Psychol. Addict. Behav..

[60] Kim M.J., Lim S.W., Kim J.H., Choe D.J., Kim J.I., Kang M.J. (2018). Effect of Mixed Fruit and Vegetable Juice on Alcohol Hangovers in Healthy Adults. Prev. Nutr. Food Sci..

[61] Central Statistical Office Population Structure by Age Since 1970. https://stat.gov.pl/obszary-tematyczne/ludnosc/ludnosc/ludnosc-piramida/.

[62] Hydes T., Gilmore W., Sheron N., Gilmore I. (2019). Treating alcohol-related liver disease from a public health perspective. J. Hepatol..

[63] Kołota A. (2018). The effect of the products of ethanol metabolism on the liver—A review. Alcohol. Drug Addict..

[64] Mondin T.C., Konradt C.E., Cardoso T.D.A., Quevedo L.D.A., Jansen K., Mattos L.D.D., Pinheiro R.T., da Silva R.A. (2013). Anxiety disorders in young people: A population-based study. Braz. J. Psychiatry.

[65] Handren L.M., Donaldson C.D., Crano W.D. (2016). Adolescent alcohol use: Protective and predictive parent, peer, and self-related factors. Prev. Sci..

[66] Public Opinion Research Center Research Report. Alcohol Consumption in Poland. https://www.cbos.pl/SPISKOM.POL/2019/K_151_19.PDF.

[67] Aiken A., Clare P.J., Wadolowski M., Hutchinson D., Najman J.M., Slade T., Bruno R., McBride N., Kypros K., Mattick R.P. (2018). Age of alcohol initiation and progression to binge drinking in adolescence: A prospective cohort study. Alcohol. Clin. Exp. Res..

[68] Chung T., Creswell K.G., Bachrach R., Clark D.B., Martin C.S. (2018). Adolescent Binge Drinking. Alcohol Res. Curr. Rev..

[69] Velleman R. Influences on How Children and Young People Learn about and Behave Towards Alcohol. https://www.jrf.org.uk/sites/default/files/jrf/migrated/files/children-alcohol-use-partone.pdf.

[70] Rowland B., Toumbourou J.W., Osborn A., Smith R., Hall J.K., Kremer P., Kelly A.B., Williams J., Leslie E. (2013). A clustered randomised trial examining the effect of social marketing and community mobilisation on the age of uptake and levels of alcohol consumption by Australian adolescents. BMJ Open.

[71] Gadalla T.M. (2012). Unhealthy behaviours among Canadian adolescents: Prevalence, trends and correlates. Chronic Dis. Inj. Can..

[72] Davoren M.P., Shiely F., Byrne M., Perry I.J. (2015). Hazardous alcohol consumption among university students in Ireland: A cross-sectional study. BMJ Open.

[73] Heather N., Partington S., Partington E., Longstaff F., Allsop S., Jankowski M., Wareham H., St Clair Gibson A. (2011). Alcohol use disorders and hazardous drinking among undergraduates at English universities. Alcohol Alcohol..

[74] Davoren M.P., Demant J., Shiely F., Perry I.J. (2016). Alcohol consumption among university students in Ireland and the United Kingdom from 2002 to 2014: A systematic review. BMC Public Health.

[75] Boniface S., Kneale J., Shelton N. (2013). Actual and perceived units of alcohol in a self-defined “usual glass” of alcoholic drinks in England. Alcohol. Clin. Exp. Res..

[76] John B., Alwyn T. (2014). Revisiting the rationale for social normative interventions in student drinking in a UK population. Addict. Behav..

[77] Black L.F., Monrouxe L.V. (2014). ‘Being sick a lot, often on each other’: Students’ alcohol-related provocation. Med. Educ..

[78] Lee C.M., Cadigan J.M., Fairlie A.M., Lewis M.A. (2018). Transitions into young adulthood: Extent to which alcohol use, perceived drinking norms, and consequences vary by education and work statuses among 18–20 year olds. Addict. Behav..

[79] Cornilov G.A., Ilkevich K.B., Shalomova E.V., Kartushina I.G., Musharatsky M.L., Mashkin N.A., Altukhov S.A. (2019). Features of alcohol consumption motives and practices by full-time and part-time training students. J. Environ. Treat. Tech..

[80] Liu X.C., Keyes K.M., Li G. (2014). Work stress and alcohol consumption among adolescents: Moderation by family and peer influences. BMC Public Health.

[81] Kim Y., Evans B.E., Hagquist C. (2019). Towards explaining time trends in adolescents’ alcohol use: A multilevel analysis of Swedish data from 1988 to 2011. Eur. J. Public Health.

[82] Beenstock J., Adams J., White M. (2011). The association between time perspective and alcohol consumption in university students: Cross sectional study. Eur. J. Public Health.

[83] Kypri K.Y.P., Cronin M., Wright C.S. (2005). Do university students drink more hazardously than their non-student peers?. Addiction.

[84] Messina M.P., D’Angelo A., Ciccarelli R., Pisciotta F., Tramonte L., Fiore M., Ferraguti G., Vitali M., Ceccanti M. (2021). Knowledge and practice towards alcohol consumption in a sample of university students. Int. J. Environ. Res. Public Health.

[85] O’Brien K.S., Ferris J., Greenlees I., Jowett S., Rhind D., Cook P.A., Kypri K. (2014). Alcohol industry sponsorship and hazardous drinking in UK university students who play sport. Addiction.

[86] Alcohol Measures for Public Health Research Alliance Report on the Impact of European Alcohol Marketing Exposure on Youth Alcohol Expectancies and Youth Drinking. https://www.drugsandalcohol.ie/19722/1/AMPHORA_WP4_longitudinal_advertising_survey.pdf.

[87] Alcohol Marketing and Young People: Time for a New Policy Agenda. https://ama.com.au/sites/default/files/documents/alcohol_marketing_young_people.pdf.

[88] Kypri K., O’Brien K., Miller P. (2009). Time for precautionary action on alcohol industry funding of sporting bodies. Addiction.

[89] Skoczylas P., Żebrowski M.R. (2009). Ocena stopnia zagrożenia alkoholizmem wśród dzieci i młodzieży ze środowisk wielkomiejskich dotkniętych patologiami społecznymi (eng. Assessment of the risk of alcoholism among children and adolescents from metropolitan environments affected by social pathologies). Probl. Hig. Epidemiol..

[90] Opielak G., Cyganok M., Zawiślak J., Putowski M., Podgórniak M., Maciejewski R. (2015). Wiek inicjacji alkoholowej u pacjentów leczonych odwykowo (eng. Age of alcohol initiation in patients treated for addiction). Curr. Probl. Psychiatry.

[91] Wojtyła A., Bojar I., Biliński P. (2010). Problem spożywania alkoholu wśród młodzieży w Polsce (eng. The problem of alcohol consumption among adolescents in Poland). Gen. Med. Health Sci..

[92] Elsayed N.M., Kim M.J., Fields K.M., Olvera R.L., Hariri A.R., Williamson D.E. (2018). Trajectories of alcohol initiation and use during adolescence: The role of stress and amygdala reactivity. J. Am. Acad. Child Adolesc. Psychiatry.

[93] Barry A.E., King J., Sears C., Harville C., Bondoc I., Joseph K. (2016). Prioritizing alcohol prevention: Establishing alcohol as the gateway drug and linking age of first drink with illicit drug use. J. Sch. Health.

[94] Tomek S., Bolland K.A., Bolland J.M., Hooper L.M., Church W.T., Bolland A.C. (2019). Age of alcohol initiation matters: Examining gender differences in the recency and frequency of alcohol use across adolescence using a sample of impoverished minority adolescents. Youth Soc..

[95] Grant B.F., Dawson D.A. (1997). Age at onset of alcohol use and its association with DSM-IV alcohol abuse and dependence: Results from the National Longitudinal Alcohol Epidemiologic Survey. J. Subst. Abus..

[96] Guttmannova K., Bailey J.A., Hill K.G., Lee J.O., Hawkins J.D., Woods M.L., Catalano R.F. (2011). Sensitive periods for adolescent alcohol use initiation: Predicting the lifetime occurrence and chronicity of alcohol problems in adulthood. J. Stud. Alcohol Drugs.

[97] Donovan J.E. (2004). Adolescent alcohol initiation: A review of psychosocial risk factors. J. Adolesc. Health.

[98] Trucco E.M. (2020). A review of psychosocial factors linked to adolescent substance use. Pharmacol. Biochem. Behav..

[99] Zeigler D.W., Wang C.C., Yoast R., Dickinson B.D., McCaffree M.A., Robinowitz C.B., Sterling M.L. (2005). The neurocognitive effects of alcohol on adolescents and college students. Prev. Med..

[100] Hingson R.W., Zha W. (2009). Age of drinking onset, alcohol use disorders, frequent heavy drinking, and unintentionally injuring oneself and others after drinking. Pediatrics.

[101] Sidorchuk A., Hemmingsson T., Romelsjo A., Allebeck P. (2012). Alcohol use in adolescence and risk of disability pension: A 39 year followup of a population-based conscription survey. PLoS ONE.

[102] Agency for Health Technology Assessment and Tariffs, Department of Health Technology Assessment Prevention of Addiction to Alcohol and Other Psychoactive Substances in Adolescents and Young Adults. https://bipold.aotm.gov.pl/assets/files/ppz/2020/RPT/12%20BIP%20RAPORT_zalec_techn_art_48aa_profilaktyka.pdf.

[103] Alessandrini G., Ciccarelli R., Battagliese G., Lombardo G., De Rosa F., Messina M.P., Vitali M., Pisciotta F., Nanut M., Attilia M.L. (2018). Treatment of alcohol dependence. Alcohol and the young: Social point of view. Riv. Psichiatr..

[104] Neves K.D.C., Teixeira M.L.D.O., Ferreira M.D.A. (2015). Factors and motivation for the consumption of alcoholic beverages in adolescence. Esc. Anna Nery.

[105] Strycker L.A., Duncan S.C., Pickering M.A. (2003). The social context of alcohol initiation among African American and White youth. J. Ethn. Subst. Abus..

[106] Osaki H., Mshana G., Mbata D., Kapiga S., Changalucha J. (2018). Social space and alcohol use initiation among youth in northern Tanzania. PLoS ONE.

[107] Martínez E.K.H., Olalde M.G.C., Aguirre A.Á. (2018). Interventions to reduce alcohol consumption in adolescents: A systematic review. Enfermería Glob..

[108] PARPA Recommendations for the Implementation and Financing of Municipal Programs for the Prevention and Resolution of Alcohol-Related Problems in 2022. https://www.parpa.pl/images/file/Rekomendacje2022.pdf.

[109] Dey M., Gmel G., Studer J., Dermota P., Mohler-Kuo M. (2013). Beverage preferences and associated drinking patterns, consequences and other substance use behaviours. Eur. J. Public Health.

[110] Jani B.D., McQueenie R., Nicholl B.I., Field R., Hanlon P., Gallacher K.I., Mair F.S., Lewsey J. (2021). Association between patterns of alcohol consumption (beverage type, frequency and consumption with food) and risk of adverse health outcomes: A prospective cohort study. BMC Med..

[111] Bergagna E., Tartaglia S. (2019). Drinking Motives, Perceived Norms, and Adolescents’ Drinking. J. Drug Issues.

[112] Kraus L., Tinghög M.E., Lindell A., Pabst A., Piontek D., Room R. (2015). Age, period and cohort effects on time trends in alcohol consumption in the Swedish adult population 1979–2011. Alcohol Alcohol..

[113] Siegel M.B., Naimi T.S., Cremeens J.L., Nelson D.E. (2011). Alcoholic beverage preferences and associated drinking patterns and risk behaviors among high school youth. Am. J. Prev. Med..

[114] Bratberg G.H., Wilsnack S., Wilsnack R., Haugland S.H., Krokstad S., Sund E.R., Bjørngaard J.H. (2016). Gender differences and gender convergence in alcohol use over the past three decades (1984–2008), The HUNT Study, Norway. BMC Public Health.

[115] Głowacz A. Spożywanie Substancji Psychoaktywnych Przez Studentów (Eng. Consumption of Psychoactive Substances by Polish Students). https://www.archaegraph.pl/lib/l231bv/Ksiega_wspolczesny-swiat_ebook-ktkerev7.pdf#page=114.

[116] Pietrzak M.J., Sienkiewicz Z., Imiela J. (2016). Spożycie alkoholu przez uczniów po 18. roku życia uczęszczających do warszawskich szkół ponadgimnazjalnych (eng. Alcohol consumption by students over 18 attending Warsaw upper secondary schools). Nurs. Probl..

[117] CBOS National Bureau for Counteracting Drug Addiction: Youth 2013. www.cinn.gov.pl.

[118] Loy J.K., Seitz N.-N., Bye E.K., Dietze P., Kilian C., Manthey J., Raitasalo K., Soellner R., Trolldal B., Törrönen J. (2021). Changes in Alcoholic Beverage Choice and Risky Drinking among Adolescents in Europe 1999–2019. Int. J. Environ. Res. Public Health.

[119] Zgliczyński W.S. (2016). Alkohol w Polsce (eng. Alcohol in Poland). Infos Socio-Econ. Issues.

[120] Cook S., De Stavola B., Saburova L., Kiryanov N., Vasiljev M., McCambridge J., McKee M., Polikina O., Gil A., Leon D.A. (2011). Socio-demographic predictors of dimensions of the AUDIT score in a population sample of working-age men in Izhevsk, Russia. Alcohol Alcohol..

[121] Akhmedjonov A., Suvankulov F. (2013). Alcohol consumption and its impact on the risk of high blood pressure in Russia. Drug Alcohol Rev..

[122] Rota M., Pelucchi C., Bertuccio P., Matsuo K., Zhang Z.F., Ito H., Hu J., Johnson K.C., Palli D., Ferraroni M. (2017). Alcohol consumption and gastric cancer risk-A pooled analysis within the StoP project consortium. Int. J. Cancer.

[123] Wotherspoon A., Elshahat S., McAlinden N., Dean K., Young I.S., Sharpe P.C., Blankenburg S., Patterson C.C., McKinley M.C., Evans A. (2020). Effect of moderate red wine versus vodka consumption on inflammatory markers related to cardiovascular disease risk: A randomized crossover study. J. Am. Coll. Nutr..

[124] Barbosa C., Cowell A.J., Dowd W.N. (2021). Alcohol consumption in response to the COVID-19 pandemic in the United States. J. Addict. Med..

[125] Ramalho R. (2020). Alcohol consumption and alcohol-related problems during the COVID-19 pandemic: A narrative review. Australas. Psychiatry.

[126] McPhee M.D., Keough M.T., Rundle S., Heath L.M., Wardell J.D., Hendershot C.S. (2020). Depression, environmental reward, coping motives and alcohol consumption during the COVID-19 pandemic. Front. Psychiatry.

[127] Schmidt R.A., Genois R., Jin J., Vigo D., Rehm J., Rush B. (2021). The early impact of COVID-19 on the incidence, prevalence, and severity of alcohol use and other drugs: A systematic review. Drug Alcohol Depend..

[128] Ahmed M.Z., Ahmed O., Aibao Z., Hanbin S., Siyu L., Ahmad A. (2020). Epidemic of COVID-19 in China and associated psychological problems. Asian J. Psychiatry.

[129] Jackson S.E., Garnett C., Shahab L., Oldham M., Brown J. (2021). Association of the COVID-19 lockdown with smoking, drinking and attempts to quit in England: An analysis of 2019–20 data. Addiction.

[130] Killgore W.D., Cloonan S.A., Taylor E.C., Lucas D.A., Dailey N.S. (2021). Alcohol dependence during COVID-19 lockdowns. Psychiatry Res..

[131] Newby J.M., O’Moore K., Tang S., Christensen H., Faasse K. (2020). Acute mental health responses during the COVID-19 pandemic in Australia. PLoS ONE.

[132] Boschuetz N., Cheng S., Mei L., Loy V.M. (2020). Changes in alcohol use patterns in the United States during COVID-19 pandemic. WMJ.

[133] Sallie S.N., Ritou V., Bowden-Jones H., Voon V. (2020). Assessing international alcohol consumption patterns during isolation from the COVID-19 pandemic using an online survey: Highlighting negative emotionality mechanisms. BMJ Open.

[134] Weerakoon S.M., Jetelina K.K., Knell G. (2020). Longer time spent at home during COVID-19 pandemic is associated with binge drinking among US adults. Am. J. Drug Alcohol Abus..

[135] Calina D., Hartung T., Mardare I., Mitroi M., Poulas K., Tsatsakis A., Rogoveanu I., Docea A.O. (2021). COVID-19 pandemic and alcohol consumption: Impacts and interconnections. Toxicol. Rep..

[136] Alcohol and COVID-19: What You Need to Know, World Health Organization Regional Office for Europe. https://www.euro.who.int/__data/assets/pdf_file/0010/437608/Alcohol-and-COVID-19-what-you-need-to-know.pdf.

[137] Zimmermann P., Curtis N. (2019). Factors that influence the immune response to vaccination. Clin. Microbiol. Rev..

[138] Dzielska A., Tabak I., Mazur J. (2013). Polish version of the DMQ-R SF questionnaire to study the motives of drinking alcohol by adolescents. Alcohol. Drug Addict..

[139] Lemańczyk R. (2019). Psychoprophylaxis of risky behaviors in adolescents. Ann. Univ. Mariae Curie-Skłodowska Sect. J..

[140] Steffens R., Sarrazin D. Guidance to Reduce Alcohol-Related Harm for Young People. Background Paper. https://www.rarha.eu/Resources/Deliverables/Lists/Work%20Package%205/Attachments/20/RARHA.WP5.T3.YoungPeople.pdf.

[141] Robinson E., Humphreys G., Jones A. (2021). Alcohol, calories, and obesity: A rapid systematic review and meta-analysis of consumer knowledge, support, and behavioral effects of energy labeling on alcoholic drinks. Obes. Rev..

[142] Hobin E., Vallance K., Zuo F., Stockwell T., Rosella L., Simniceanu A., Hammond D. (2018). Testing the efficacy of alcohol labels with standard drink information and national drinking guidelines on consumers’ ability to estimate alcohol consumption. Alcohol Alcohol..

[143] McNally K., Noonan L.L., Cameron M., Phillips K., Baidoobonso S., Sabapathy D. (2019). Public awareness of low-risk alcohol use guidelines. Health Promot. Pract..

[144] Vallance K., Stockwell T., Zhao J., Shokar S., Schoueri-Mychasiw N., Hammond D., Greenfield T.K., McGavock J., Weerasinghe A., Hobin E. (2020). Baseline assessment of alcohol-related knowledge of and support for alcohol warning labels among alcohol consumers in Northern Canada and associations with key sociodemographic characteristics. J. Stud. Alcohol Drugs.

[145] Buykx P., Li J., Gavens L., Hooper L., Lovatt M., Gomes de Matos E., Meier P., Holmes J. (2016). Public awareness of the link between alcohol and cancer in England in 2015: A population-based survey. BMC Public Health.

[146] Kaźmierczak M., Gierszewska M., Mieczkowska E., Gebuza G., Wróbel-Bania A., Szumotalska H. (2016). Ocena wiedzy kobiet na temat alkoholowego zespołu płodowego oraz narażenia płodu na ekspozycję alkoholu. (eng. Assessment of women’s knowledge about Foetal Alcohol Syndrome and foetal alcohol exposure). Educ. Saf..

[147] Bouchery E.E., Harwood H.J., Sacks J.J., Simon C.J., Brewer R.D. (2011). Economic costs of excessive alcohol consumption in the US, 2006. Am. J. Prev. Med..

[148] Mohapatra S., Patra J., Popova S., Duhig A., Rehm J. (2010). Social cost of heavy drinking and alcohol dependence in high-income countries. Int. J. Public Health.

