# Supplementary material for: Assessment of the Prevalence of Alcoholic Beverage Consumption and Knowledge of the Impact of Alcohol on Health in a Group of Polish Young Adults Aged 18–35: A Cross-Sectional Study
Source: Int J Environ Res Public Health. 2022 Nov 22;19(23):15425. doi: 10.3390/ijerph192315425 (PMC9737381; doi:10.3390/ijerph192315425)
Supplement: Supplementary file 1 [file ijerph-19-15425-s001.zip › ijerph-1907782-supplementary.pdf]

International Journal of Environmental Research and Public Health

MDPI

Table S1. The questionnaire used in the study.

**Characteristics of the group**	
**Sex** *(closed question, single choice)*<br>Male<br>Female	**Age**<br>*Open question*
**Weight**<br>*Open question*	**Height**<br>*Open question*
**Marital status** *(closed question, single choice)*<br>Single<br>Married<br>Divorced<br>Widowed	**Size of the place of residence** *(closed question, single choice)*<br>Village<br>City up to 5,000 residents<br>City over 5,000 up to 50 thousand residents<br>City over 50,000 up to 200 thousand residents<br>City over 200,000 residents
**Profession** *(closed question, single choice)*<br>Student<br>Full-time job<br>Odd job<br>Unemployed	**Do you suffer from any chronic diseases / allergies?** *If so, please list.*<br>*Pytanie otwarte*
**Are you pregnant or breastfeeding?** *(closed question, single choice)*<br>Yes<br>No<br>Not applicable	**Have you ever been diagnosed with alcohol dependence?** *(closed question, single choice)*<br>Yes<br>No
**Questions about alcohol consumption**	
**Have you ever consumed alcoholic beverages?** *(closed question, single choice)*<br>Yes<br>No	**At what age have you consciously consumed a drink containing alcohol for the first time?** *(closed question, single choice)*<br>Up to 10 years<br>From 10 to 15 years old<br>15-18 years<br>Over 18 years of age
**Where was the first consumption of alcoholic beverages?** *(closed question, single choice)*<br>Family meeting<br>With friends<br>In the absence of parents at home<br>School trip<br>School party<br>Party<br>Other	**What kind of alcoholic beverage did you drink first?** *(closed question, single choice)*<br>Beer<br>Wine<br>Champagne<br>Vodka<br>Tincture<br>Whiskey<br>Other
**How often do you drink alcoholic beverages?** *(closed question, single choice)*<br>I do not drink<br>Once a month or less<br>2 to 4 times a month<br>2 to 3 times a week<br>4 times a week or more	**How many portions of alcoholic beverages (e.g. a glass of vodka, a can of beer) do you drink on one occasion?** *(closed question, single choice)*<br>I don't drink alcohol<br>1-2 portions<br>3-4 portions<br>5-6 portions<br>7,8 or 9 portions<br>10 or more portions

**How often do you drink 6 or more alcohol drinks on one occasion?** *(closed question, single choice)*<br>Never<br>Less often than once a month<br>Once a month<br>Once a week<br>Every day or almost every day	**What type of alcoholic beverages do you most often consume currently?** *(closed question, single choice)*<br>Beer<br>Wine<br>Champagne<br>Vodka<br>Tincture<br>Whiskey<br>Other
**How often did you drink beer during the last 12 months before the test?** *(closed question, single choice)*<br>I have not drunk<br>1-2x<br>Less than 1x a month<br>More than 1x a month<br>Every day	**How often did you drink wine in the last 12 months before the test?** *(closed question, single choice)*<br>I have not drunk<br>1-2x<br>Less than 1x a month<br>More than 1x a month<br>Every day
**How often did you drink spirits (vodka, whiskey, tincture, liqueur, etc.) in the last 12 months before the test?** *(closed question, single choice)*<br>I have not drunk<br>1-2x<br>Less than 1x a month<br>More than 1x a month<br>Every day	**How often did you drink "coloured" alcoholic beverages (drinks, coloured shots) in the last 12 months before the test?** *(closed question, single choice)*<br>I have not drunk<br>1-2x<br>Less than 1x a month<br>More than 1x a month<br>Every day
**How often did you drink beer during the last 30 days before the test?** *(closed question, single choice)*<br>I have not drunk<br>1-2x<br>Less than 1x a week<br>More than 1x a week<br>Every day	**How often did you drink wine in the last 30 days before the test?** *(closed question, single choice)*<br>I have not drunk<br>1-2x<br>Less than 1x a week<br>More than 1x a week<br>Every day
**How often did you drink spirits (vodka, whiskey, tincture, liqueur, etc.) in the last 30 days before the test?** *(closed question, single choice)*<br>I have not drunk<br>1-2x<br>Less than 1x a week<br>More than 1x a week<br>Every day	**How often did you drink "coloured" alcoholic beverages (drinks, colored shots) in the last 30 days before the test?** *(closed question, single choice)*<br>I have not drunk<br>1-2x<br>Less than 1x a week<br>More than 1x a week<br>Every day
**What amount of beer do you most often consume on one occasion?** *(closed question, single choice)*<br>I have not drunk in the last 12 months<br>Less than one bottle or can (less than 0.5 liters)<br>1-2 typical bottles or cans (over 0.5 to 1 liter)<br>3-4 typical bottles or cans (from 1 liter to 2 liters)<br>More than 4 typical bottles or cans (over 2 liters)	**What amount of wine do you most often consume on one occasion?** *(closed question, single choice)*<br>I have not drunk wine in the last 12 months<br>Less than one glass (less than 100 grams)<br>1-3 glasses (from 100 grams to 300 grams)<br>More than 3 glasses, but less than a bottle (300 grams to 750 grams)<br>A bottle or more (750 grams or more)
**What amount of spirits (vodka, whiskey, tincture, liqueur, etc.) do you most often consume on one occasion?** *(closed question, single choice)*<br>I have not drunk any spirits in the last 12 months<br>Less than one glass (less than 50 grams)<br>1-2 glasses (from 50 grams to 100 grams)	**What amount of "coloured" alcoholic beverages (drinks, colored shots) do you most often consume on one occasion?** *(closed question, single choice)*<br>I have not drunk "colored" alcoholic beverages in the last 12 months<br>Less than one glass (less than 200 grams)

3-5 glasses (from 100 grams to 250 grams)<br>More than 5 glasses (250 grams or more)	1-3 glasses (200 to 600 grams)<br>More than 3 glasses (600 grams or more)
**Has your drinking habits changed since the start of the COVID-19 pandemic?** *(closed question, single choice)*<br>I drink less<br>I drink more<br>I drink a different type of alcohol - with a higher alcohol content (e.g. vodka)<br>I drink a different type of alcohol - with less alcohol content (e.g. beer)<br>It has not changed	
**Questions about the style of drinking alcohol**	
**How much money do you spend per month on alcoholic beverages?** *(closed question, single choice)*<br>I don't drink alcohol<br>I don't buy alcohol, others offer me<br>Up to 12 USD<br>12-24 USD<br>Above 24 USD	**Why do you drink alcoholic beverages?** (*closed question, multiple choice)*<br>Because friends drink<br>To forget about troubles<br>To have fun, be in a better mood<br>To take courage<br>To kill boredom<br>It is tasty<br>Out of curiosity<br>Other
**Have you ever been drunk? If so, how many times?** *(closed question, single choice)*<br>It has not happened<br>Once<br>Several times<br>Repeatedly	**How often during the last year have you been unable to remember what happened the night before because of your drinking?** *(closed question, single choice)*<br>Never<br>Less often than once a month<br>Once a month<br>Once a week<br>Every day or almost every day
**During the last year, how often have you done something inappropriate because of drinking alcohol, which would violate the norms and customs adopted in your environment?** *(closed question, single choice)*<br>Never<br>Less often than once a month<br>Once a month<br>Once a week<br>Every day or almost every day	**During the last year, how often have you found that you cannot stop drinking once you have started drinking?** *(closed question, single choice)*<br>Never<br>Less often than once a month<br>Once a month<br>Once a week<br>Every day or almost every day
**During the last year, how often have you had to drink in the morning to recover from the "heavy drinking" you had the day before?** *(closed question, single choice)*<br>Never<br>Less often than once a month<br>Once a month<br>Once a week<br>Every day or almost every day	**During the last year, how often have you experienced guilt or remorse after drinking alcohol?** *(closed question, single choice)*<br>Never<br>Less often than once a month<br>Once a month<br>Once a week<br>Every day or almost every day
**Have you or any other person ever been injured as a result of drinking alcohol?** *(closed question, single choice)*<br>Yes, but not in the last year<br>Yes, in the last year<br>No	**Has a relative, friend or doctor ever had an interest in or suggested restricting alcohol consumption?** *(closed question, single choice)*<br>Yes, but not in the last year<br>Yes, in the last year<br>No
**Where do you get information on the impact of alcohol on human health from?** (*closed question, multiple choice)*<br>Internet<br>Television	

Radio
Own observation
Press
Teachers
Parents
Peers
Books
Healthcare workers
Other

Table S2. Knowledge test on the effects of ethyl alcohol on health.

**What kind of alcohol is in alcoholic beverages?** *(closed question, single choice)*<br>Ethanol (1)<br>Methanol (0)<br>Propanol (0)<br>All listed depending on the type of alcohol (0)	**Have you ever heard the term "standard drink" in relation to alcohol?** *(closed question, single choice)*<br>Yes (1)<br>No (0)<br>I do not know (0)
**How many "standard drink" are in 250 ml of 5% beer?** *(closed question, single choice)*<br>0,5 (0)<br>1 (1)<br>1,5 (0)<br>2 (0)<br>I do not know (0)	**How many "standard drink" are in 30 ml of 40% vodka?** *(closed question, single choice)*<br>1 (1)<br>2 (0)<br>3 (0)<br>4 (0)<br>I do not know (0)
**How many "standard drink" are in 100 ml of 12% wine?** *(closed question, single choice)*<br>0,5 (0)<br>1 (1)<br>1,5 (0)<br>2 (0)<br>I do not know (0)	**What is the maximum amount of "standard drink" of alcohol with low health risk for a man?** *(closed question, single choice)*<br>1 (0)<br>2-3 (0)<br>4 (1)<br>6 or moreI do not know (0)
**What is the maximum amount of "standard drink" of alcohol with low health risk for a woman?** *(closed question, single choice)*<br>1 (0)<br>2-3 (1)<br>4 (0)<br>6 or more (0)<br>I do not know (0)	**How much calories does 1g of ethyl alcohol have?** *(closed question, single choice)*<br>0 kcal (0)<br>2 kcal (0)<br>4 kcal (0)<br>7 kcal (1)<br>9 kcal (0)<br>I do not know (0)
**How much calories does a typical can of beer (500 ml, 4.5% alcohol) have?** *(closed question, single choice)*<br>124 kcal (0)<br>200 kcal (0)<br>245 kcal (1)<br>340 kcal (0)	**How many calories does a typical glass of semi-sweet red wine (120 ml, 14% alcohol) have?** *(closed question, single choice)*<br>82 kcal (0)<br>101 kcal (0)<br>115 kcal (1)<br>135 kcal (0)
**How much calories does a typical glass of vodka have (25 ml, 40% alcohol)?** *(closed question, single choice)*<br>55 kcal (1)<br>79 kcal (0)<br>162 kcal (0)<br>210 kcal (0)	**How many calories does a typical "Mojito" drink (200ml) have?** *(closed question, single choice)*<br>77 kcal (0)<br>125 kcal (1)<br>224 kcal (0)<br>340 kcal (0)
**In your opinion, can you become addicted to alcohol while drinking only beer?** *(closed question, single choice)*<br>Yes (1)<br>No (0)	**Do you think that regular drinking of small doses of alcohol can lead to addiction?** *(closed question, single choice)*<br>Yes (1)<br>No (0)

I do not know (0)	I do not know (0)

Do you think alcohol can be treated as a therapeutic agent (headaches, colds, malaise)? *(closed question, single choice)*
Yes (0)
No (1)
I do not know (0)

Do you think the following sentences are true or false? (*question with options: true, false, don't know)*
Drinking milk before drinking an alcoholic drink will slow down the absorption of alcohol in your body. (T/F/DN = 1)
Alcohol is classified as a stimulant. (F = 1)
It takes about as many hours to fully burn the consumed alcohol as the glasses to drink. (F = 1)
Alcohol abuse shortens life expectancy by about 10 years. (P = 1)
Consumption of alcohol during pregnancy has no effect on the fetus. (F = 1)
Excessive alcohol consumption may increase the risk of colon cancer. (P = 1)
Beer strengthens the heart and lowers blood pressure (T/F/DN = 1)
Beer cleans the kidneys. (T/F/DN = 1)
Polyphenols contained in wine inhibit the development of atherosclerosis. (P = 1)
The "hangover" lasts up to 20 hours and begins after the body has rid the blood of the alcohol (P = 1)
Alcohol dehydrates the body (P = 1)
The resveratrol contained in wine does not have a major impact on health due to its low concentration. (P = 1)
Tomato juice can reduce hangover discomfort. (P = 1)

Table S3. Cronbach's alpha values for the subtests of the questionnaire.

**Part of the questionnaire**	**Alpha**
The frequency and style of consumption of alcoholic beverages	0,7125
The AUDIT test	0,7727
The knowledge test	0,8019

Table S4. The results of the AUDIT test in the study group divided according to the risk of drinking alcohol.

**Parameter**	**Total (*n* = 480)**	**Low level of risk (*n* = 320)**	**Risky consumption of ethyl alcohol (*n* = 160)**	***p***
How often do you drink alcoholic beverages?				
I do not drink	29 (6.0%)	28 (8.8%)	1 (0.6%)	
Once a month or less	120 (25.0%)	108 (33.8%)	12 (7.5%)	
2 to 4 times a month	212 (44.2%)	143 (44.7%)	69 (43.1%)	<0.0001
2 to 3 times a week	94 (19.6%)	38 (11.9%)	56 (35.0%)	
4 times a week or more	25 (5.2%)	3 (0.9%)	22 (13.8%)	
How many portions of alcoholic beverages (e.g. a glass of vodka, a can of beer) do you drink on one occasion?				
I don't drink alcohol	29 (6.0%)	28 (8.8%)	1 (0.6%)	
1-2 portions	176 (36.7%)	147 (45.9%)	29 (18.1%)	
3-4 portions	157 (32.7%)	110 (34.4%)	47 (29.4%)	<0.0001
5-6 portions	60 (12.5%)	24 (7.5%)	36 (22.5%)	
7,8 or 9 portions	27 (5.6%)	8 (2.5%)	19 (11.9%)	
10 or more portions	31 (6.5%)	3 (0.9%)	28 (17.5%)	
How often do you drink 6 or more alcohol drinks on one occasion?				
Never				
Less often than once a	122 (25.4%)	118 (36.9%)	4 (2.5%)	
month	228 (47.5%)	178 (55.6%)	50 (31.3%)	
Once a month	84 (17.5%)	24 (7.5%)	60 (37.5%)	<0.0001
Once a week	44 (9.2%)	0 (0.0%)	44 (27.5%)	
Every day or almost every day	2 (0.4%)	0 (0.0%)	2 (1.3%)	
How often during the last year have you been unable to remember what happened the night before because of your drinking?				
Never				
Less often than once a	330 (68.7%)	285 (89.1%)	45 (28.1%)	
month	113 (23.5%)	35 (10.9%)	78 (48.8%)	
Once a month	23 (4.8%)	0 (0.0%)	23 (14.4%)	<0.0001
Once a week	10 (2.1%)	0 (0.0%)	10 (6.3%)	
Every day or almost every day	4 (0.8%)	0 (0.0%)	4 (2.5%)	
During the last year, how often have you done something inappropriate because of drinking alcohol, which would violate the norms and customs adopted in your environment?				
Never				
Less often than once a	343 (71.5%)	288 (90.0%)	55 (34.4%)	
month	117 (24.4%)	31 (9.7%)	86 (53.8%)	
Once a month	13 (2.7%)	1 (0.3%)	12 (7.5%)	<0.0001
Once a week	7 (1.4%)	0 (0.0%)	7 (4.4%)	
Every day or almost every day	0 (0.0%)	0 (0.0%)	0 (0.0%)	

During the last year, how often have you found that you cannot stop drinking once you have started drinking?				
Never	392 (81.6%)	309 (96.6%)	83 (51.9%)	
Less often than once a month	49 (10.2%)	11 (3.4%)	38 (23.8%)	
Once a month	25 (5.2%)	0 (0.0%)	25 (15.6%)	<0.0001
Once a week	12 (2.5%)	0 (0.0%)	12 (7.5%)	
Every day or almost every day	2 (0.4%)	0 (0.0%)	2 (1.3%)	
During the last year, how often have you had to drink in the morning to recover from the "heavy drinking" you had the day before?				
Never	404 (84.2%)	311 (97.2%)	93 (53.1%)	
Less often than once a month	40 (8.3%)	8 (2.5%)	32 (20.0%)	
Once a month	17 (3.5%)	1 (0.3%)	16 (10.0%)	<0.0001
Once a week	17 (3.5%)	0 (0.0%)	17 (10.6%)	
Every day or almost every day	2 (0.4%)	0 (0.0%)	2 (1.3%)	
During the last year, how often have you experienced guilt or remorse after drinking alcohol?				
Never	286 (59.6%)	248 (77.5%)	38 (23.8%)	
Less often than once a month	140 (23.2%)	69 (21.6%)	71 (44.4%)	
Once a month	37 (7.7%)	3 (0.9%)	34 (21.3%)	<0.0001
Once a week	14 (2.9%)	0 (0.0%)	14 (8.8%)	
Every day or almost every day	3 (0.6%)	0 (0.0%)	3 (1.9%)	
Have you or any other person ever been injured as a result of drinking alcohol?				
Yes, but not in the last year	68 (14.2%)	27 (8.4%)	41 (25.6%)	
Yes, in the last year	32 (6.6%)	3 (0.9%)	29 (18.1%)	<0.0001
No	380 (79.2%)	290 (90.6%)	90 (56.3%)	
Has a relative, friend or doctor ever had an interest in or suggested restricting alcohol consumption?				
Yes, but not in the last year	37 (7.7%)	12 (3.8%)	25 (15.6%)	
Yes, in the last year	34 (7.1%)	1 (0.3%)	33 (20.6%)	<0.0001
No	409 (85.2%)	307 (95.9%)	102 (63.8%)	

Values express counts (*n*) and percentages (%). Statistically significant differences between the risk of drinking alcohol were analysed using Pearson's chi-square ($\chi 2$).

Table S5. Age and place of first contact with alcoholic beverages and the type of alcoholic beverages drunk for the first time in the study group divided according to the risk of drinking alcohol.

**Parameter**	**Total (*n* = 480)**	**Low level of risk (*n* = 320)**	**Risky consumption of ethyl alcohol (*n* = 160)**	***p***
At what age have you consciously consumed a drink containing alcohol for the first time?				
Up to 10 years	17 (3.5%)	10 (3.1%)	7 (4.4%)	<0.0001
From 10 to 15 years old	162 (33.8%)	88 (27.5%)	74 (46.3%)	
15-18 years	257 (53.5%)	184 (57.5%)	73 (45.6%)	
Over 18 years of age	44 (9.2%)	38 (11.9%)	6 (3.8%)	
Where was the first consumption of alcoholic beverages?				
Family meeting	110 (22.9%)	85 (26.6%)	25 (15.6%)	0.0344
With friends	139 (28.9%)	76 (23.8%)	63 (39.4%)	
In the absence of parents at home	47 (9.8%)	27 (8.4%)	20 (12.5%)	
School trip	24 (5.0%)	16 (5.0%)	8 (5.0%)	
School party	24 (5.0%)	14 (4.4%)	10 (6.3%)	
Party	135 (28.1%)	102 (31.9%)	33 (20.6%)	
Other	1 (0.2%)	0 (0.0%)	1 (0.6%)	
What kind of alcoholic beverage did you drink first?				
Beer	270 (56.3%)	175 (54.7%)	95 (59.4%)	0.0214
Wine	58 (12.1%)	46 (14.4%)	12 (20.7%)	
Champagne	35 (7.3%)	30 (9.4%)	5 (3.1%)	
Vodka	89 (18.5%)	51 (15.9%)	38 (23.8%)	
Tincture	18 (3.7%)	11 (3.4%)	7 (4.4%)	
Whiskey	3 (0.6)	3 (0.9%)	0 (0.0%)	
Other				
- Liqueur	1 (0.2)	0 (0.0%)	1 (0.6%)	
- Drink	4 (0.8)	2 (0.6%)	2 (1.3%)	
- Cider	2 (0.4)	2 (0.6%)	0 (0.0%)	

Values express counts (n) and percentages (%). Statistically significant differences between the risk of drinking alcohol were analysed using Pearson's chi-square (χ2).

Table S6. Alcoholic beverage consumption habits in the study group divided according to the risk of drinking alcohol.

Parameter	Total (*n* = 480)	Low level of risk (*n* = 320)	Risky consumption of ethyl alcohol (*n* = 160)	*p*
What type of alcoholic beverages do you most often consume currently?				
Beer	215 (44.8%)	135 (42.2%)	80 (50.0%)	0.0001
Wine	135 (28.1%)	111 (34.7%)	24 (15.0%)	
Champagne	5 (1.0%)	4 (1.3%)	1 (0.6%)	
Vodka	49 (10.2%)	17 (5.3%)	32 (20.0%)	
Tincture	11 (2.3%)	9 (2.8%)	2 (1.3%)	
Whiskey	41 (8.5%)	26 (8.1%)	15 (9.4%)	
Other	12 (2.5%)	6 (1.9%)	6 (3.7%)	
None	12 (2.5%)	12 (3.8%)	0 (0.0%)	
How much money do you spend per month on alcoholic beverages?				
I don't drink alcohol	26 (5.4%)	25 (7.8%)	1 (0.6%)	<0.0001
I don't buy alcohol, others offer me	66 (13.8%)	58 (18.1%)	8 (5.0%)	
Up to 12 USD	243 (50.6%)	178 (55.6%)	65 (40.6%)	
12-24 USD	88 (18.3%)	44 (13.8%)	44 (27.5%)	
Above 24 USD	57 (11.9%)	15 (4.7%)	42 (26.3%)	
Have you ever been drunk? If so, how many times?				
It has not happened	60 (12.5%)	60 (18.8%)	0 (0.0%)	<0.0001
Once	49 (10.2%)	42 (13.1%)	7 (4.4%)	
Several times	204 (42.5%)	150 (46.9%)	54 (33.8%)	
Repeatedly	167 (34.8%)	68 (21.3%)	99 (61.9%)	
Has your drinking habits changed since the start of the COVID-19 pandemic?				
I drink less	126 (26.2%)	90 (28.1%)	36 (22.5%)	0.0025
I drink more	54 (11.3%)	25 (7.8%)	29 (18.1%)	
I drink a different type of alcohol - with a higher alcohol content (e.g. vodka)	22 (4.6%)	9 (2.8%)	13 (8.1%)	
I drink a different type of alcohol - with less alcohol content (e.g. beer)	23 (4.8%)	19 (5.9%)	4 (2.5%)	
It has not changed	255 (53.1%)	177 (55.3%)	78 (48.8%)	

Values express counts (n) and percentages (%). Statistically significant differences between the risk of drinking alcohol were analysed using Pearson's chi-square ($\chi2$).

Table S7. The frequency of consumption of various types of alcoholic beverages in the last 12 months in the study group divided according to the risk of drinking alcohol.

Parameter	Total (n = 480)	Low level of risk (n = 320)	Risky consumption of ethyl alcohol (n = 160)	p
How often did you drink beer during the last 12 months before the test?				
I have not drunk	53 (11.0%)	49 (15.3%)	4 (2.5%)	<0.0001
1-2x	59 (12.3%)	49 (15.3%)	10 (6.3%)	
Less than 1x a month	138 (28.8%)	107 (33.4%)	31 (19.4%)	
More than 1x a month	221 (46.0%)	114 (35.6%)	107 (66.9%)	
Every day	9 (1.9%)	1 (0.3%)	8 (5.0%)	
How often did you drink wine in the last 12 months before the test?				
I have not drunk	94 (19.6%)	69 (21.6%)	25 (15.6%)	0.1576
1-2x	105 (21.9%)	73 (22.8%)	32 (20.0%)	
Less than 1x a month	155 (32.3%)	103 (32.2%)	52 (32.5%)	
More than 1x a month	126 (26.3%)	75 (23.4%)	51 (31.9%)	
Every day	0 (0.0%)	0 (0.0%)	0 (0.0%)	
How often did you drink spirit drinks (vodka, whiskey, tincture, liqueur, etc.) in the last 12 months before the test?				
I have not drunk	90 (18.8%)	83 (25.9%)	7 (4.4%)	<0.0001
1-2x	99 (20.6%)	86 (26.9%)	13 (8.1%)	
Less than 1x a month	165 (34.4%)	107 (33.4%)	58 (36.3%)	
More than 1x a month	124 (25.8%)	43 (13.4%)	81 (50.6%)	
Every day	2 (0.4%)	1 (0.3%)	1 (0.6%)	
How often did you drink "coloured" alcoholic beverages (drinks, colored shots) in the last 12 months before the test?				
I have not drunk	130 (27.1%)	111 (34.7%)	19 (11.9%)	<0.0001
1-2x	121 (25.2%)	85 (26.6%)	36 (22.5%)	
Less than 1x a month	150 (31.3%)	92 (28.8%)	58 (36.3%)	
More than 1x a month	78 (16.2%)	31 (9.7%)	47 (29.4%)	
Every day	1 (0.2%)	1 (0.3%)	0 (0.0%)	

Values express counts (n) and percentages (%). Statistically significant differences between the risk of drinking alcohol were analysed using Pearson's chi-square ($\chi 2$).

Table S8. Frequency of consumption of various types of alcoholic beverages over the last 30 days in the study group divided according to the risk of drinking alcohol.

**Parameter**	**Total (*n* = 480)**	**Low level of risk (*n* = 320)**	**Risky consumption of ethyl alcohol (*n* = 160)**	***p***
How often did you drink beer during the last 30 days before the test?				
I have not drunk	127 (26.5%)	109 (34.1%)	18 (11.3%)	
1-2x	127 (26.5%)	95 (29.7%)	32 (20.0%)	
Less than 1x a week	121 (25.2%)	79 (24.7%)	42 (26.3%)	<0.0001
More than 1x a week	96 (20.0%)	36 (11.3%)	60 (37.5%)	
Every day	9 (1.8%)	1 (0.3%)	8 (5.0%)	
How often did you drink wine in the last 30 days before the test?				
I have not drunk	226 (47.1%)	158 (49.4%)	68 (42.5%)	
1-2x	130 (27.0%)	84 (26.3%)	46 (28.8%)	
Less than 1x a week	103 (21.5%)	69 (21.6%)	34 (21.3%)	0.1097
More than 1x a week	20 (4.2%)	9 (2.8%)	11 (6.9%)	
Every day	1 (0.2%)	0 (0.0%)	1 (0.6%)	
How often did you drink spirit drinks (vodka, whiskey, tincture, liqueur, etc.) in the last 30 days before the test?				
I have not drunk	219 (45.6%)	189 (59.1%)	30 (18.8%)	
1-2x	122 (25.4%)	74 (23.1%)	48 (30.0%)	
Less than 1x a week	102 (21.3%)	49 (15.3%)	53 (33.1%)	<0.0001
More than 1x a week	35 (7.3%)	7 (2.2%)	28 (17.5%)	
Every day	2 (0.4%)	1 (0.3%)	1 (0.6%)	
How often did you drink "colored" alcoholic beverages (drinks, colored shots) in the last 30 days before the test?				
I have not drunk	274 (57.1%)	212 (66.3%)	62 (38.7%)	
1-2x	110 (22.9%)	68 (21.3%)	42 (26.3%)	
Less than 1x a week	75 (15.6%)	34 (10.6%)	41 (25.6%)	<0.0001
More than 1x a week	21 (4.4%)	6 (1.9%)	15 (9.4%)	
Every day	0 (0.0%)	0 (0.0%)	0 (0.0%)	

Values express counts (n) and percentages (%). Statistically significant differences between the risk of drinking alcohol were analysed using Pearson's chi-square ($\chi 2$).

Table S9. The average amount of alcoholic beverages consumed at one time in the study group divided according to the risk of drinking alcohol.

Parameter	Total (*n* = 480)	Low level of risk (*n* = 320)	Risky consumption of ethyl alcohol (*n* = 160)	*p*
What amount of beer do you most often consume on one occasion?				
I have not drunk in the last 12 months	48 (10.0%)	44 (13.8%)	4 (2.5%)	<0.0001
Less than one bottle or can (less than 0.5 liters)	63 (13.1%)	53 (16.6%)	10 (6.3%)	
1-2 typical bottles or cans (over 0.5 to 1 liter)	268 (55.8%)	189 (59.1%)	79 (49.4%)	
3-4 typical bottles or cans (from 1 liter to 2 liters)	78 (16.3%)	31 (9.7%)	47 (29.4%)	
More than 4 typical bottles or cans (over 2 liters)	23 (4.8%)	3 (0.9%)	20 (12.5%)	
What amount of wine do you most often consume on one occasion?				
I have not drunk wine in the last 12 months	91 (18.9%)	64 (20.0%)	27 (16.9%)	0.0007
Less than one glass (less than 100 grams)	54 (11.3%)	38 (11.9%)	16 (10.0%)	
1-3 glasses (from 100 grams to 300 grams)	247 (51.5%)	173 (54.1%)	74 (46.3%)	
More than 3 glasses, but less than a bottle (300 grams to 750 grams)	57 (11.9%)	35 (10.9%)	22 (13.8%)	
A bottle or more (750 grams or more)	31 (6.4%)	10 (3.1%)	21 (13.1%)	
What amount of spirit drinks (vodka, whiskey, tincture, liqueur, etc.) do you most often consume on one occasion?				
I have not drunk any spirits in the last 12 months	93 (19.4%)	85 (26.6%)	8 (5.0%)	<0.0001
Less than one glass (less than 50 grams)	32 (6.7%)	26 (8.1%)	6 (3.8%)	
1-2 glasses (from 50 grams to 100 grams)	100 (20.8%)	82 (25.6%)	18 (11.3%)	
3-5 glasses (from 100 grams to 250 grams)	97 (20.2%)	67 (20.9%)	30 (18.8%)	
More than 5 glasses (250 grams or more)	158 (32.9%)	60 (18.8%)	98 (61.3%)	
What amount of "colored" alcoholic beverages (drinks, colored shots) do you most often consume on one occasion?				
I have not drunk "colored" alcoholic beverages in the last 12 months	140 (29.2%)	113 (35.3%)	27 (16.9%)	<0.0001
Less than one glass (less than 200 grams)	65 (13.5%)	44 (13.8%)	21 (13.1%)	
1-3 glasses (200 to 600 grams)	203 (42.3%)	136 (42.5%)	67 (41.9%)	
More than 3 glasses (600 grams or more)	72 (15.0%)	27 (8.4%)	45 (28.1%)	

Values express counts (n) and percentages (%). Statistically significant differences between the risk of drinking alcohol were analysed using Pearson's chi-square ($\chi 2$).

Table S10. The results of the knowledge test on the impact of ethyl alcohol consumption on health in the studied group divided according to the risk of drinking alcohol.

Parameter	Total (*n* = 480)	Low level of risk (*n* = 320)	Risky consumption of ethyl alcohol (*n* = 160)	*p*
What kind of alcohol is in alcoholic beverages?				
Ethanol	408 (85.0%)	276 (86.3%)	132 (82.5%)	0.3975
Methanol	17 (3.5%)	9 (2.8%)	8 (5.0%)	
Propanol	0 (0.0%)	0 (0.0%)	0 (0.0%)	
All listed depending on the type of alcohol	55 (11.5%)	35 (10.9%)	20 (12.5%)	
Have you ever heard the term "standard drink" in relation to alcohol?				
Yes	180 (35.5%)	122 (38.1%)	58 (36.3%)	0.0213
No	215 (44.8%)	152 (47.5%)	63 (39.4%)	
I do not know	85 (17.7%)	46 (14.4%)	39 (24.4%)	
How many "standard drink" are in 250 ml of 5% beer?				
0,5	87 (18.1%)	52 (16.3%)	35 (21.9%)	0.2276
1	114 (23.7%)	74 (23.1%)	40 (25.0%)	
1,5	6 (1.3%)	6 (1.9%)	0 (0.0%)	
2	12 (2.5%)	8 (2.5%)	4 (2.5%)	
I do not know	261 (54.4%)	180 (56.3%)	81 (50.6%)	
How many "standard drink" are in 30 ml of 40% vodka?				
1	186 (38.7%)	114 (35.6%)	72 (45.0%)	0.2033
2	23 (4.8%)	18 (5.6%)	5 (3.1%)	
3	10 (2.1%)	6 (1.9%)	4 (2.5%)	
4	2 (0.4%)	2 (0.6%)	0 (0.0%)	
I do not know	259 (53.9%)	180 (56.3%)	79 (49.4%)	
How many "standard drink" are in 100 ml of 12% wine?				
0,5	36 (7.5%)	24 (7.5%)	12 (7.5%)	0.2054
1	146 (30.4%)	88 (27.5%)	58 (36.3%)	
1,5	10 (2.1%)	6 (1.9%)	4 (2.5%)	
2	9 (1.9%)	8 (2.5%)	1 (0.6%)	
I do not know	279 (58.1%)	194 (60.6%)	85 (53.1%)	
What is the maximum amount of "standard drink" of alcohol with low health risk for a man?				
1	31 (6.5%)	23 (7.2%)	8 (5.0%)	0.1211
2-3	83 (17.3%)	55 (17.2%)	28 (17.5%)	
4	80 (16.6%)	49 (15.3%)	31 (19.4%)	
6 or more	29 (6.0%)	14 (4.4%)	15 (9.4%)	
I do not know	257 (53.5%)	179 (55.9%)	78 (48.8%)	
What is the maximum amount of "standard drink" of alcohol with low health risk for a woman?				
1	73 (15.2%)	51 (15.9%)	22 (13.8%)	0.0923
2-3	113 (23.5%)	73 (22.8%)	40 (25.0%)	
4	28 (5.8%)	15 (4.7%)	13 (8.1%)	
6 or more	7 (1.4%)	2 (0.6%)	5 (3.1%)	
I do not know	259 (53.9%)	179 (55.9%)	80 (50.0%)	
How much calories does 1g of ethyl alcohol have?				
0 kcal	15 (3.1%)	8 (2.5%)	7 (4.4%)	0.8621
2 kcal	20 (4.2%)	12 (3.8%)	8 (5.0%)	
4 kcal	51 (10.6%)	34 (10.6%)	17 (10.6%)	
7 kcal	136 (28.3%)	93 (29.1%)	43 (26.9%)	

9 kcal	36 (7.5%)	25 (7.8%)	11 (6.9%)	
I do not know	222 (46.2%)	148 (46.3%)	74 (46.3%)	
How much calories does a typical can of beer (500 ml, 4.5% alcohol) have?				
124 kcal	23 (4.8%)	13 (4.1%)	10 (6.3%)	0.1233
200 kcal	89 (18.5%)	62 (19.4%)	27 (16.9%)	
245 kcal	230 (47.9%)	144 (45.0%)	86 (53.8%)	
340 kcal	138 (28.7%)	101 (31.6%)	37 (23.1%)	
How many calories does a typical glass of semi-sweet red wine (120 ml, 14% alcohol) have?				
82 kcal	67 (13.9%)	41 (12.8%)	26 (16.3%)	0.3790
101 kcal	124 (24.8%)	79 (24.7%)	45 (28.1%)	
115 kcal	170 (35.4%)	114 (35.6%)	56 (35.0%)	
135 kcal	119 (24.8%)	86 (26.9%)	33 (20.6%)	
How much calories does a typical glass of vodka have (25 ml, 40% alcohol)?				
55 kcal	179 (37.3%)	111 (34.7%)	68 (42.5%)	0.2285
79 kcal	177 (36.9%)	121 (37.8%)	56 (35.0%)	
162 kcal	76 (15.8%)	51 (15.9%)	25 (15.6%)	
210 kcal	48 (10.0%)	37 (11.6%)	11 (6.9%)	
How many calories does a typical "Mojito" drink (200ml) have?				
77 kcal	20 (4.2%)	9 (2.8%)	11 (6.9%)	0.1671
125 kcal	104 (21.6%)	67 (20.9%)	37 (23.1%)	
224 kcal	191 (39.8%)	130 (40.6%)	61 (38.1%)	
340 kcal	165 (34.4%)	114 (35.6%)	51 (31.9%)	
In your opinion, can you become addicted to alcohol while drinking only beer?				
Yes	444 (92.5%)	307 (95.9%)	137 (85.6%)	<0.0001
No	17 (3.5%)	4 (1.3%)	13 (8.1%)	
I do not know	19 (3.9%)	9 (2.8%)	10 (6.3%)	
Do you think that regular drinking of small doses of alcohol can lead to addiction?				
Yes	407 (84.8%)	282 (88.1%)	125 (78.1%)	0.0034
No	54 (11.3%)	25 (7.8%)	29 (18.1%)	
I do not know	19 (3.9%)	13 (4.1%)	6 (3.8%)	
Do you think alcohol can be treated as a therapeutic agent (headaches, colds, malaise)?				
Yes	119 (24.8%)	60 (18.8%)	59 (36.9%)	<0.0001
No	326 (67.9%)	240 (75.0%)	86 (53.8%)	
I do not know	35 (7.3%)	20 (6.3%)	15 (9.4%)	
Drinking milk before drinking an alcoholic drink will slow down the absorption of alcohol in your body.				
Truth	54 (11.3%)	35 (10.9%)	19 (11.9%)	0.9282
False	224 (46.6%)	151 (47.2%)	73 (45.6%)	
I do not know	202 (42.1%)	134 (41.9%)	68 (42.5%)	
Alcohol is classified as a stimulant.				
Truth	267 (55.6%)	173 (54.1%)	94 (58.8%)	0.2005
False	139 (28.9%)	91 (28.4%)	48 (30.0%)	
I do not know	74 (15.4%)	56 (17.5%)	18 (11.3%)	
It takes about as many hours to fully burn the consumed alcohol as the glasses to drink.				
Truth	86 (17.9%)	50 (15.6%)	36 (22.5%)	0.0121
False	207 (43.1%)	131 (40.9%)	76 (47.5%)	
I do not know	187 (39.0%)	139 (43.4%)	48 (30.0%)	
Alcohol abuse shortens life expectancy by about 10 years.				
Truth	325 (67.7%)	214 (66.9%)	111 (69.4%)	0.5640
False	46 (9.6%)	29 (9.1%)	17 (10.6%)	
I do not know	109 (22.7%)	77 (24.1%)	32 (20.0%)	

Consumption of alcohol during pregnancy has no effect on the fetus.				
Truth	28 (5.8%)	16 (5.0%)	12 (7.5%)	
False	443 (92.3%)	300 (93.8%)	143 (89.4%)	0.1860
I do not know	9 (1.9%)	4 (1.3%)	5 (3.1%)	
Excessive alcohol consumption may increase the risk of colon cancer.				
Truth	377 (78.5%)	256 (80.0%)	121 (75.6%)	
False	15 (3.1%)	7 (2.2%)	8 (5.0%)	0.2120
I do not know	88 (18.3%)	57 (17.8%)	31 (19.4%)	
Beer strengthens the heart and lowers blood pressure.				
Truth	44 (9.2%)	19 (5.9%)	25 (15.6%)	
False	330 (68.7%)	230 (71.9%)	100 (62.5%)	0.0022
I do not know	106 (22.1%)	71 (22.2%)	35 (21.9%)	
Beer cleans the kidneys.				
Truth	263 (54.8%)	165 (51.6%)	98 (61.3%)	
False	154 (32.1%)	114 (35.6%)	40 (25.0%)	0.0593
I do not know	63 (13.1%)	41 (12.8%)	22 (13.8%)	
Polyphenols contained in wine inhibit the development of atherosclerosis.				
Truth	232 (48.3%)	150 (46.9%)	82 (51.3%)	
False	79 (16.5%)	58 (18.1%)	21 (13.1%)	0.3580
I do not know	169 (35.2%)	112 (35.0%)	57 (35.6%)	
The "hangover" lasts up to 20 hours and begins after the body has rid the blood of the alcohol				
Truth	165 (34.4%)	105 (32.8%)	60 (37.5%)	
False	154 (32.1%)	95 (29.7%)	59 (36.9%)	0.0320
I do not know	161 (33.5%)	120 (37.5%)	41 (25.6%)	
Alcohol dehydrates the body				
Truth	437 (91.0%)	286 (89.4%)	151 (94.4%)	
False	18 (3.8%)	14 (4.4%)	4 (2.5%)	0.1927
I do not know	25 (5.2%)	20 (6.3%)	5 (3.1%)	
The resveratrol contained in wine does not have a major impact on health due to its low concentration.				
Truth	77 (16.0%)	56 (17.5%)	21 (13.1%)	
False	131 (27.3%)	93 (29.1%)	38 (23.8%)	0.1260
I do not know	272 (56.7%)	171 (53.4%)	101 (63.1%)	
Tomato juice can reduce hangover discomfort.				
Truth	268 (55.8%)	174 (54.4%)	94 (58.8%)	
False	52 (10.8%)	31 (9.7%)	21 (13.1%)	0.1753
I do not know	160 (33.3%)	115 (35.9%)	45 (28.1%)	

Values express counts (n) and percentages (%). Statistically significant differences between the risk of drinking alcohol were analysed using Pearson's chi-square ($\chi 2$).

Table S11. The results of the AUDIT test in the study group divided according to the period of data collection.

Parameter	Total (n = 480)	Period I* (n = 141)	Period II** (n = 339)	P***
How often do you drink alcoholic beverages?				
I do not drink	29 (6.0%)	0 (0.0%)	29 (8.5%)	
Once a month or less	120 (25.0%)	36 (25.5%)	84 (24.8%)	
2 to 4 times a month	212 (44.2%)	68 (48.2%)	144 (42.5%)	0.0050
2 to 3 times a week	94 (19.6%)	32 (22.7%)	62 (18.3%)	
4 times a week or more	25 (5.2%)	5 (3.5%)	20 (5.9%)	
How many portions of alcoholic beverages (e.g. a glass of vodka, a can of beer) do you drink on one occasion?				
I don't drink alcohol	29 (6.0%)	1 (0.7%)	28 (8.3%)	
1-2 portions	176 (36.7%)	58 (41.1%)	118 (34.8%)	
3-4 portions	157 (32.7%)	47 (33.3%)	110 (32.5%)	0.0067
5-6 portions	60 (12.5%)	12 (8.5%)	48 (14.6%)	
7,8 or 9 portions	27 (5.6%)	11 (7.8%)	16 (4.7%)	
10 or more portions	31 (6.5%)	12 (8.5%)	19 (5.6%)	
How often do you drink 6 or more alcohol drinks on one occasion?				
Never				
Less often than once a	122 (25.4%)	29 (20.6%)	93 (27.4%)	
month	228 (47.5%)	74 (52.5%)	154 (45.4%)	
Once a month	84 (17.5%)	24 (17.0%)	60 (17.7%)	0.5046
Once a week	44 (9.2%)	13 (9.2%)	31 (9.1%)	
Every day or almost every	2 (0.4%)	1 (0.7%)	1 (0.3%)	
day				
How often during the last year have you been unable to remember what happened the night before because of your drinking?				
Never				
Less often than once a	330 (68.7%)	93 (65.9%)	237 (69.9%)	
month	113 (23.5%)	41 (29.1%)	72 (21.2%)	
Once a month	23 (4.8%)	4 (2.8%)	19 (5.6%)	0.3000
Once a week	10 (2.1%)	2 (1.4%)	8 (2.4%)	
Every day or almost every	4 (0.8%)	1 (0.7%)	3 (0.9%)	
day				
During the last year, how often have you done something inappropriate because of drinking alcohol, which would violate the norms and customs adopted in your environment?				
Never				
Less often than once a	343 (71.5%)	100 (70.9%)	243 (71.7%)	
month	117 (24.4%)	36 (25.5%)	81 (23.9%)	
Once a month	13 (2.7%)	4 (2.8%)	9 (2.6%)	0.8259
Once a week	7 (1.4%)	1 (0.7%)	6 (1.8%)	
Every day or almost every	0 (0.0%)	0 (0.0%)	0 (0.0%)	
day				
During the last year, how often have you found that you cannot stop drinking once you have started drinking?				
Never	392 (81.6%)	121 (85.8%)	271 (79.9%)	
Less often than once a	49 (10.2%)	17 (12.0%)	32 (9.4%)	
month	25 (5.2%)	1 (0.7%)	24 (7.1%)	0.0187
Once a month	12 (2.5%)	1 (0.7%)	11 (3.2%)	
Once a week	2 (0.4%)	1 (0.7%)	1 (0.3%)	

Every day or almost every day				
During the last year, how often have you had to drink in the morning to recover from the "heavy drinking" you had the day before?				
Never	404 (84.2%)	127 (90.1%)	277 (81.7%)	
Less often than once a month	40 (8.3%)	9 (6.4%)	31 (9.1%)	
Once a month	17 (3.5%)	4 (2.8%)	13 (3.8%)	0.1174
Once a week	17 (3.5%)	1 (0.7%)	16 (4.7%)	
Every day or almost every day	2 (0.4%)	0 (0.0%)	2 (0.6%)	
During the last year, how often have you experienced guilt or remorse after drinking alcohol?				
Never	286 (59.6%)	86 (60.9%)	200 (59.0%)	
Less often than once a month	140 (23.2%)	43 (30.5%)	97 (28.6%)	
Once a month	37 (7.7%)	5 (3.5%)	32 (9.4%)	0.2049
Once a week	14 (2.9%)	6 (4.3%)	8 (2.3%)	
Every day or almost every day	3 (0.6%)	1 (0.7%)	2 (0.6%)	
Have you or any other person ever been injured as a result of drinking alcohol?				
Yes, but not in the last year	68 (14.2%)	17 (12.0%)	51 (15.0%)	
Yes, in the last year	32 (6.6%)	12 (8.5%)	20 (5.9%)	0.4388
No	380 (79.2%)	112 (79.4%)	268 (79.1%)	
Has a relative, friend or doctor ever had an interest in or suggested restricting alcohol consumption?				
Yes, but not in the last year	37 (7.7%)	5 (3.5%)	32 (9.4%)	
Yes, in the last year	34 (7.1%)	7 (4.9%)	27 (7.9%)	0.0355
No	409 (85.2%)	129 (91.5%)	280 (82.6%)	

* the period from June to September 2021, ** the period from March to April 2022, *** Values express counts (*n*) and percentages (%).Statistically significant differences between the groups of the two recruitment periods were analysed using the Pearson's chi-square (χ2).

Table S12. Drinking risk groups based on the AUDIT test results divided according to the period of data collection.

Interpretation	Total (*n* = 480)	Period I (*n* = 141)	Period II (*n* = 339)	*p*
Low-risk consumption	320 (66.7%)	95 (67.4%)	225 (66.4%)	0.8317
High-risk alcohol consumption	160 (33.3%)	46 (32.6%)	114 (33.6%)	

Values express counts (*n*) and percentages (%).Statistically significant differences between the groups of the two recruitment periods were analysed using the Pearson's chi-square (χ2).

Table S13. Age and place of first contact with alcoholic beverages and the type of alcoholic beverages drunk for the first time in the study group divided according to the period of data collection.

Parameter	Total (*n* = 480)	Period I (*n* = 141)	Period II (*n* = 339)	*p*
At what age have you consciously consumed a drink containing alcohol for the first time?				
Up to 10 years	17 (3.5%)	4 (2.8%)	13 (3.8%)	0.1907
From 10 to 15 years old	162 (33.8%)	49 (34.8%)	113 (33.3%)	
15-18 years	257 (53.5%)	81 (57.5%)	176 (51.9%)	
Over 18 years of age	44 (9.2%)	7 (4.9%)	37 (10.9%)	
Where was the first consumption of alcoholic beverages?				
Family meeting	110 (22.9%)	35 (24.8%)	75 (22.1%)	0.1787
With friends	139 (28.9%)	40 (28.4%)	99 (29.2%)	
In the absence of parents at home	47 (9.8%)	12 (8.5%)	35 (10.3%)	
School trip	24 (5.0%)	4 (2.8%)	20 (5.9%)	
School party	24 (5.0%)	9 (6.4%)	16 (4.7%)	
Party	135 (28.1%)	45 (31.9%)	90 (26.5%)	
Other	1 (0.2%)	1 (0.7%)	0 (0.0%)	
What kind of alcoholic beverage did you drink first?				
Beer	270 (56.3%)	82 (58.2%)	188 (55.5%)	0.6082
Wine	58 (12.1%)	12 (8.5%)	46 (13.6%)	
Champagne	35 (7.3%)	8 (5.7%)	27 (7.9%)	
Vodka	89 (18.5%)	34 (24.1%)	55 (16.2%)	
Tincture	18 (3.7%)	2 (1.4%)	16 (4.7%)	
Whiskey	3 (0.6)	1 (0.7%)	2 (0.6%)	
Other				
- Liqueur	1 (0.2)	0 (0.0%)	1 (0.3%)	
- Drink	4 (0.8)	1 (0.7%)	3 (0.9%)	
- Cider	2 (0.4)	1 (0.7%)	1 (0.3%)	

Values express counts (*n*) and percentages (%).Statistically significant differences between the groups of the two recruitment periods were analysed using the Pearson's chi-square (χ2).

Table S14. Alcoholic beverage consumption habits in the study group divided according to the period of data collection.

Parameter	Total (*n* = 480)	Period I (*n* = 141)	Period II (*n* = 339)	*p*
What type of alcoholic beverages do you most often consume currently?				
Beer	215 (44.8%)	73 (51.8%)	142 (41.9%)	0.5558
Wine	135 (28.1%)	37 (26.2%)	98 (28.9%)	
Champagne	5 (1.0%)	0 (0.0%)	5 (1.5%)	
Vodka	49 (10.2%)	13 (9.2%)	36 (10.6%)	
Tincture	11 (2.3%)	2 (1.4%)	9 (2.6%)	
Whiskey	41 (8.5%)	13 (9.2%)	28 (8.3%)	
Other	12 (2.5%)	0 (0.0%)	12 (3.5%)	
None	12 (2.5%)	3 (2.1%)	9 (2.6%)	
How much money do you spend per month on alcoholic beverages?				
I don't drink alcohol	26 (5.4%)	0 (0.0%)	26 (7.6%)	0.0148
I don't buy alcohol, others offer me	66 (13.8%)	18 (12.8%)	48 (14.2%)	
Up to 12 USD	243 (50.6%)	75 (53.2%)	168 (49.6%)	
12-24 USD	88 (18.3%)	28 (19.9%)	60 (17.7%)	
Above 24 USD	57 (11.9%)	20 (14.2%)	37 (10.9%)	
Have you ever been drunk? If so, how many times?				
It has not happened	60 (12.5%)	13 (9.2%)	47 (13.9%)	0.1297
Once	49 (10.2%)	19 (13.5%)	30 (8.8%)	
Several times	204 (42.5%)	54 (38.3%)	150 (44.2%)	
Repeatedly	167 (34.8%)	55 (39.0%)	112 (33.0%)	
Has your drinking habits changed since the start of the COVID-19 pandemic?				
I drink less	126 (26.2%)	37 (26.2%)	88 (25.9%)	0.3822
I drink more	54 (11.3%)	17 (12.0%)	36 (10.6%)	
I drink a different type of alcohol - with a higher alcohol content (e.g. vodka)	22 (4.6%)	6 (4.2%)	18 (5.3%)	
I drink a different type of alcohol - with less alcohol content (e.g. beer)	23 (4.8%)	2 (1.4%)	22 (6.5%)	
It has not changed	255 (53.1%)	79 (56.0%)	175 (51.6%)	

Values express counts (*n*) and percentages (%).Statistically significant differences between the groups of the two recruitment periods were analysed using the Pearson's chi-square (χ2).

Table S15. The frequency of consumption of various types of alcoholic beverages in the last 12 months in the study group divided according to the period of data collection.

Parameter	Total (*n* = 480)	Period I (*n* = 141)	Period II (*n* = 339)	*p*
How often did you drink beer during the last 12 months before the test?				
I have not drunk	53 (11.0%)	7 (4.9%)	46 (13.6%)	0.0005
1-2x	59 (12.3%)	8 (5.6%)	51 (15.0%)	
Less than 1x a month	138 (28.8%)	53 (37.6%)	85 (25.1%)	
More than 1x a month	221 (46.0%)	70 (49.6%)	151 (44.5%)	
Every day	9 (1.9%)	3 (2.1%)	6 (1.8%)	
How often did you drink wine in the last 12 months before the test?				
I have not drunk	94 (19.6%)	23 (16.3%)	71 (20.9%)	0.4891
1-2x	105 (21.9%)	28 (19.9%)	77 (22.7%)	
Less than 1x a month	155 (32.3%)	50 (35.5%)	105 (30.9%)	
More than 1x a month	126 (26.3%)	40 (28.4%)	86 (25.4%)	
Every day	0 (0.0%)	0 (0.0%)	0 (0.0%)	
How often did you drink spirit drinks (vodka, whiskey, tincture, liqueur, etc.) in the last 12 months before the test?				
I have not drunk	90 (18.8%)	20 (14.2%)	70 (20.6%)	0.2050
1-2x	99 (20.6%)	25 (17.7%)	74 (21.8%)	
Less than 1x a month	165 (34.4%)	55 (39.0%)	110 (32.5%)	
More than 1x a month	124 (25.8%)	41 (29.1%)	83 (24.5%)	
Every day	2 (0.4%)	0 (0.0%)	2 (0.6%)	
How often did you drink "coloured" alcoholic beverages (drinks, coloured shots) in the last 12 months before the test?				
I have not drunk	130 (27.1%)	35 (24.8%)	95 (28.0%)	0.5984
1-2x	121 (25.2%)	38 (26.9%)	83 (24.5%)	
Less than 1x a month	150 (31.3%)	49 (34.7%)	101 (29.8%)	
More than 1x a month	78 (16.2%)	19 (13.5%)	59 (17.4%)	
Every day	1 (0.2%)	0 (0.0%)	1 (0.3%)	

Values express counts (n) and percentages (%).Statistically significant differences between the groups of the two recruitment periods were analysed using the Pearson’s chi-square (χ2).

Table S16. Frequency of consumption of various types of alcoholic beverages over the last 30 days in the study group divided according to the period of data collection.

Parameter	Total (*n* = 480)	Period I (*n* = 141)	Period II (*n* = 339)	*p*
How often did you drink beer during the last 30 days before the test?				
I have not drunk	127 (26.5%)	22 (15.6%)	105 (30.9%)	0.0002
1-2x	127 (26.5%)	43 (30.5%)	84 (24.8%)	
Less than 1x a week	121 (25.2%)	31 (22.0%)	90 (26.5%)	
More than 1x a week	96 (20.0%)	43 (30.5%)	53 (15.6%)	
Every day	9 (1.8%)	2 (1.4%)	7 (2.0%)	
How often did you drink wine in the last 30 days before the test?				
I have not drunk	226 (47.1%)	63 (44.7%)	163 (48.1%)	0.1267
1-2x	130 (27.0%)	32 (22.7%)	98 (28.9%)	
Less than 1x a week	103 (21.5%)	38 (26.9%)	65 (19.2%)	
More than 1x a week	20 (4.2%)	7 (4.9%)	13 (3.8%)	
Every day	1 (0.2%)	1 (0.7%)	0 (0.0%)	
How often did you drink spirit drinks (vodka, whiskey, tincture, liqueur, etc.) in the last 30 days before the test?				
I have not drunk	219 (45.6%)	57 (40.4%)	162 (47.8%)	0.3435
1-2x	122 (25.4%)	43 (3045%)	79 (23.3%)	
Less than 1x a week	102 (21.3%)	29 (2046%)	73 (21.5%)	
More than 1x a week	35 (7.3%)	12 (845%)	23 (6.8%)	
Every day	2 (0.4%)	0 (040%)	2 (0.6%)	
How often did you drink "coloured" alcoholic beverages (drinks, coloured shots) in the last 30 days before the test?				
I have not drunk	274 (57.1%)	74 (52.5%)	200 (59.0%)	0.2290
1-2x	110 (22.9%)	41 (29.1%)	69 (20.4%)	
Less than 1x a week	75 (15.6%)	20 (14.2%)	55 (16.2%)	
More than 1x a week	21 (4.4%)	6 (4.3%)	15 (4.4%)	
Every day	0 (0.0%)	0 (0.0%)	0 (0.0%)	

Values express counts (n) and percentages (%).Statistically significant differences between the groups of the two recruitment periods were analysed using the Pearson's chi-square ($\chi 2$).

Table S17. The average amount of alcoholic beverages consumed at one time in the study group divided according to the period of data collection.

Parameter	Total (*n* = 480)	Period I (*n* = 141)	Period II (*n* = 339)	*p*
What amount of beer do you most often consume on one occasion?				
I have not drunk in the last 12 months	48 (10.0%)	8 (5.7%)	40 (11.8%)	0.3398
Less than one bottle or can (less than 0.5 liters)	63 (13.1%)	21 (14.9%)	42 (12.4%)	
1-2 typical bottles or cans (over 0.5 to 1 liter)	268 (55.8%)	80 (56.7%)	188 (55.5%)	
3-4 typical bottles or cans (from 1 liter to 2 liters)	78 (16.3%)	25 (17.7%)	53 (15.6%)	
More than 4 typical bottles or cans (over 2 liters)	23 (4.8%)	7 (4.9%)	16 (4.7%)	
What amount of wine do you most often consume on one occasion?				
I have not drunk wine in the last 12 months	91 (18.9%)	23 (16.3%)	68 (20.1%)	0.8878
Less than one glass (less than 100 grams)	54 (11.3%)	17 (12.0%)	37 (10.9%)	
1-3 glasses (from 100 grams to 300 grams)	247 (51.5%)	75 (53.2%)	172 (50.7%)	
More than 3 glasses, but less than a bottle (300 grams to 750 grams)	57 (11.9%)	16 (11.3%)	41 (12.1%)	
A bottle or more (750 grams or more)	31 (6.4%)	10 (7.1%)	21 (6.2%)	
What amount of spirit drinks (vodka, whiskey, tincture, liqueur, etc.) do you most often consume on one occasion?				
I have not drunk any spirits in the last 12 months	93 (19.4%)	22 (15.6%)	71 (20.9%)	0.5308
Less than one glass (less than 50 grams)	32 (6.7%)	9 (6.4%)	23 (6.8%)	
1-2 glasses (from 50 grams to 100 grams)	100 (20.8%)	27 (19.1%)	73 (21.5%)	
3-5 glasses (from 100 grams to 250 grams)	97 (20.2%)	33 (23.4%)	64 (18.9%)	
More than 5 glasses (250 grams or more)	158 (32.9%)	50 (35.5%)	108 (31.9%)	
What amount of "coloured" alcoholic beverages (drinks, coloured shots) do you most often consume on one occasion?				
I have not drunk "coloured" alcoholic beverages in the last 12 months	140 (29.2%)	37 (26.2%)	103 (30.4%)	0.7591
Less than one glass (less than 200 grams)	65 (13.5%)	19 (13.5%)	46 (13.6%)	
1-3 glasses (200 to 600 grams)	203 (42.3%)	61 (43.3%)	142 (41.9%)	
More than 3 glasses (600 grams or more)	72 (15.0%)	24 (17.0%)	48 (14.1%)	

Values express counts (n) and percentages (%).Statistically significant differences between the groups of the two recruitment periods were analysed using the Pearson’s chi-square (χ2).

Table S18. Reasons for drinking alcohol in the study group divided according to the period of data collection.

Parameter	Total (*n* = 480)	Period I (*n* = 141)	Period II (*n* = 339)	*p*
Because friends drink	174 (36.2%)	41 (29.1%)	133 (39.2%)	0.0350
To forget about troubles	67 (13.9%)	23 (16.3%)	44 (12.9%)	0.3372
To have fun, be in a better mood	286 (59.6%)	96 (68.1%)	190 (56.0%)	0.0144
To take courage	58 (12.1%)	17 (12.1%)	41 (12.1%)	0.9908
To kill boredom	74 (15.4%)	19 (13.5%)	55 (16.2%)	0.4475
It is tasty	227 (47.3%)	84 (59.6%)	143 (42.2%)	0.0005
Out of curiosity	28 (5.8%)	9 (6.4%)	19 (5.6%)	0.7404
Other				
- Type of work	2 (0.4%)	1 (0.7%)	1 (0.3%)	0.5211
- Support sleep	1 (0.2%)	0 (0.0%)	1 (0.3%)	0.5185
- I don't drink alcohol	9 (1.9%)	0 (0.0%)	9 (2.7%)	0.0508

Values express counts (n) and percentages (%).Statistically significant differences between the groups of the two recruitment periods were analysed using the Pearson's chi-square ($\chi2$).

Table S19. The results of the knowledge test on the impact of ethyl alcohol consumption on health in the studied group divided according to the period of data collection.

Parameter	Total (*n* = 480)	Period I (*n* = 141)	Period II (*n* = 339)	*p*
What kind of alcohol is in alcoholic beverages?				
Ethanol	408 (85.0%)	130 (92.2%)	278 (82.0%)	0.0059
Methanol	17 (3.5%)	5 (3.5%)	12 (3.5%)	
Propanol	0 (0.0%)	0 (0.0%)	0 (0.0%)	
All listed depending on the type of alcohol	55 (11.5%)	6 (4.2%)	49 (14.5%)	
Have you ever heard the term "standard drink" in relation to alcohol?				
Yes	180 (35.5%)	44 (31.2%)	136 (40.1%)	0.0825
No	215 (44.8%)	65 (46.1%)	150 (44.2%)	
I do not know	85 (17.7%)	32 (22.7%)	53 (15.6%)	
How many "standard drink" are in 250 ml of 5% beer?				
0,5	87 (18.1%)	28 (19.8%)	59 (17.4%)	0.5338
1	114 (23.7%)	35 (24.8%)	79 (23.3%)	
1,5	6 (1.3%)	0 (0.0%)	6 (1.8%)	
2	12 (2.5%)	4 (2.8%)	8 (2.3%)	
I do not know	261 (54.4%)	74 (52.5%)	187 (55.2%)	
How many "standard drink" are in 30 ml of 40% vodka?				
1	186 (38.7%)	54 (38.3%)	132 (38.9%)	0.3023
2	23 (4.8%)	10 (7.1%)	13 (3.8%)	
3	10 (2.1%)	1 (0.7%)	9 (2.6%)	
4	2 (0.4%)	0 (0.0%)	2 (0.6%)	
I do not know	259 (53.9%)	76 (53.9%)	183 (53.9%)	
How many "standard drink" are in 100 ml of 12% wine?				
0,5	36 (7.5%)	11 (7.8%)	25 (7.4%)	0.9527
1	146 (30.4%)	43 (30.5%)	103 (30.4%)	
1,5	10 (2.1%)	2 (1.4%)	8 (2.3%)	
2	9 (1.9%)	2 (1.4%)	7 (2.0%)	

I do not know	279 (58.1%)	83 (58.9%)	196 (57.8%)	
What is the maximum amount of "standard drink" of alcohol with low health risk for a man?				
1	31 (6.5%)	6 (4.2%)	25 (7.4%)	
2-3	83 (17.3%)	20 (14.2%)	63 (18.6%)	
4	80 (16.6%)	28 (19.9%)	52 (15.3%)	0.2103
6 or more	29 (6.0%)	12 (8.5%)	17 (5.0%)	
I do not know	257 (53.5%)	75 (53.2%)	182 (53.7%)	
What is the maximum amount of "standard drink" of alcohol with low health risk for a woman?				
1	73 (15.2%)	18 (12.8%)	55 (16.2%)	
2-3	113 (23.5%)	32 (22.7%)	81 (23.9%)	
4	28 (5.8%)	9 (6.4%)	19 (5.6%)	0.4602
6 or more	7 (1.4%)	4 (2.8%)	3 (0.9%)	
I do not know	259 (53.9%)	78 (55.3%)	181 (53.4%)	
How much calories does 1g of ethyl alcohol have?				
0 kcal	15 (3.1%)	2 (1.4%)	13 (3.8%)	
2 kcal	20 (4.2%)	9 (6.4%)	11 (3.2%)	
4 kcal	51 (10.6%)	13 (9.2%)	38 (11.2%)	
7 kcal	136 (28.3%)	44 (31.2%)	92 (27.1%)	0.3009
9 kcal	36 (7.5%)	8 (5.6%)	28 (8.2%)	
I do not know	222 (46.2%)	65 (46.1%)	157 (46.3%)	
How much calories does a typical can of beer (500 ml, 4.5% alcohol) have?				
124 kcal	23 (4.8%)	7 (4.9%)	16 (4.7%)	
200 kcal	89 (18.5%)	23 (16.3%)	66 (19.5%)	0.7073
245 kcal	230 (47.9%)	73 (51.8%)	157 (46.3%)	
340 kcal	138 (28.7%)	38 (26.9%)	100 (29.5%)	
How many calories does a typical glass of semi-sweet red wine (120 ml, 14% alcohol) have?				
82 kcal	67 (13.9%)	24 (17.0%)	43 (12.7%)	
101 kcal	124 (24.8%)	35 (24.8%)	89 (26.2%)	0.3729
115 kcal	170 (35.4%)	53 (37.6%)	117 (34.5%)	
135 kcal	119 (24.8%)	29 (20.6%)	90 (26.5%)	
How much calories does a typical glass of vodka have (25 ml, 40% alcohol)?				
55 kcal	179 (37.3%)	47 (33.3%)	132 (38.9%)	
79 kcal	177 (36.9%)	67 (47.5%)	110 (32.4%)	0.0130
162 kcal	76 (15.8%)	18 (12.8%)	58 (17.1%)	
210 kcal	48 (10.0%)	9 (6.4%)	39 (11.5%)	
How many calories does a typical "Mojito" drink (200ml) have?				
77 kcal	20 (4.2%)	2 (1.4%)	18 (5.3%)	
125 kcal	104 (21.6%)	30 (21.3%)	74 (21.8%)	0.2713
224 kcal	191 (39.8%)	58 (41.1%)	133 (39.2%)	
340 kcal	165 (34.4%)	51 (36.2%)	114 (33.6%)	
In your opinion, can you become addicted to alcohol while drinking only beer?				
Yes	444 (92.5%)	134 (95.0%)	310 (91.5%)	
No	17 (3.5%)	3 (2.1%)	14 (4.1%)	0.3867
I do not know	19 (3.9%)	4 (2.8%)	15 (4.4%)	
Do you think that regular drinking of small doses of alcohol can lead to addiction?				
Yes	407 (84.8%)	130 (92.2%)	277 (81.7%)	
No	54 (11.3%)	7 (4.9%)	47 (13.8%)	0,0115
I do not know	19 (3.9%)	4 (2.8%)	15 (4.4%)	
Do you think alcohol can be treated as a therapeutic agent (headaches, colds, malaise)?				
Yes	119 (24.8%)	38 (26.9%)	81 (23.9%)	0.1170

No	326 (67.9%)	98 (69.5%)	228 (67.3%)	
I do not know	35 (7.3%)	5 (3.5%)	30 (8.8%)	
Drinking milk before drinking an alcoholic drink will slow down the absorption of alcohol in your body.				
Truth	54 (11.3%)	17 (1.,0%)	37 (10.9%)	
False	224 (46.6%)	69 (48.9%)	155 (45.7%)	0.6754
I do not know	202 (42.1%)	55 (39.0%)	147 (43.4%)	
Alcohol is classified as a stimulant.				
Truth	267 (55.6%)	82 (58.2%)	185 (54.6%)	
False	139 (28.9%)	40 (28.4%)	99 (29.2%)	0.6900
I do not know	74 (15.4%)	19 (13.5%)	55 (16.2%)	
It takes about as many hours to fully burn the consumed alcohol as the glasses to drink.				
Truth	86 (17.9%)	17 (12.0%)	69 (20.3%)	
False	207 (43.1%)	68 (48.2%)	139 (41.0%)	0.0796
I do not know	187 (39.0%)	56 (39.7%)	131 (38.6%)	
Alcohol abuse shortens life expectancy by about 10 years.				
Truth	325 (67.7%)	95 (67.4%)	230 (67.8%)	
False	46 (9.6%)	16 (11.3%)	30 (8.8%)	0.6597
I do not know	109 (22.7%)	30 (21.3%)	79 (23.3%)	
Consumption of alcohol during pregnancy has no effect on the fetus.				
Truth	28 (5.8%)	7 (4.9%)	21 (6.2%)	
False	443 (92.3%)	131 (92.9%)	312 (92.0%)	0.8459
I do not know	9 (1.9%)	3 (2.1%)	6 (1.8%)	
Excessive alcohol consumption may increase the risk of colon cancer.				
Truth	377 (78.5%)	112 (79.4%)	265 (78.2%)	
False	15 (3.1%)	4 (2.8%)	11 (3.2%)	0.9452
I do not know	88 (18.3%)	25 (17.7%)	63 (18.6%)	
Beer strengthens the heart and lowers blood pressure.				
Truth	44 (9.2%)	5 (3.5%)	39 (11.5%)	
False	330 (68.7%)	103 (7.,0%)	227 (66.9%)	0.0226
I do not know	106 (22.1%)	33 (23.4%)	73 (21.5%)	
Beer cleans the kidneys.				
Truth	263 (54.8%)	80 (56.7%)	183 (53.9%)	
False	154 (32.1%)	45 (31.9%)	109 (32.1%)	0.7333
I do not know	63 (13.1%)	16 (11.3%)	47 (13.9%)	
Polyphenols contained in wine inhibit the development of atherosclerosis.				
Truth	232 (48.3%)	64 (45.4%)	168 (49.6%)	
False	79 (16.5%)	27 (19.2%)	52 (15.3%)	0.5381
I do not know	169 (35.2%)	50 (35.5%)	119 (35.1%)	
The "hangover" lasts up to 20 hours and begins after the body has rid the blood of the alcohol				
Truth	165 (34.4%)	55 (39.0%)	110 (32.4%)	
False	154 (32.1%)	52 (36.9%)	102 (30.1%)	0.0186
I do not know	161 (33.5%)	34 (24.1%)	127 (37.5%)	
Alcohol dehydrates the body				
Truth	437 (91.0%)	128 (90.8%)	309 (91.2%)	
False	18 (3.8%)	8 (5.7%)	10 (2.9%)	0.2197
I do not know	25 (5.2%)	5 (3.5%)	20 (5.9%)	
The resveratrol contained in wine does not have a major impact on health due to its low concentration.				
Truth	77 (16.0%)	17 (12.0%)	60 (17.7%)	
False	131 (27.3%)	34 (24.1%)	97 (28.6%)	0.1042
I do not know	272 (56.7%)	90 (63.8%)	182 (53.7%)	

Tomato juice can reduce hangover discomfort.				
Truth	268 (55.8%)	90 (63.8%)	178 (52.5%)	
False	52 (10.8%)	7 (4.9%)	45 (13.3%)	0.0116
I do not know	160 (33.3%)	44 (31.2%)	116 (34.2%)	

Values express counts (n) and percentages (%).Statistically significant differences between the groups of the two recruitment periods were analysed using the Pearson's chi-square ($\chi 2$). .

Table S20. The level of knowledge about ethyl alcohol in the study group divided according to the period of data collection.

Interpretation	Total (*n* = 480)	Period I (*n* = 141)	Period II (*n* = 339)	*P*
High knowledge index	42 (8.8%)	9 (6.4%)	33 (9.7%)	
Average knowledge index	407 (59.4%)	130 (92.2%)	277 (81.7%)	0.0055
Low knowledge index	31 (31.8%)	2 (1.4%)	29 (8.6%)	

Values express counts (n) and percentages (%).Statistically significant differences between the groups of the two recruitment periods were analysed using the Pearson's chi-square (χ2).

Table S21. Knowledge sources in the study group divided according to the period of data collection.

Parameter	Total (*n* = 480)	Period I (*n* = 141)	Period II (*n* = 339)	*p*
Internet	384 (80.0%)	114 (80.8%)	270 (79.6%)	0.7637
Television	75 (15.6%)	21 (14.9%)	54 (15.9%)	0.7759
Radio	22 (4.6%)	2 (1.4%)	20 (5.9%)	0.0325
Own observation	354 (73.7%)	102 (72.3%)	252 (74.3%)	0.6518
Press	37 (7.7%)	11 (7.8%)	26 (7.7%)	0.9607
Teachers	93 (19.4%)	31 (22.0%)	62 (18.3%)	0.3506
Parents	99 (20.6%)	29 (20.6%)	70 (20.6%)	0.9839
Peers	196 (40.8%)	58 (41.1%)	138 (40.7%)	0.9309
Books	71 (14.8%)	22 (15.6%)	49 (14.4%)	0.7468
Healthcare workers	78 (16.3%)	20 (14.2%)	58 (17.1%)	0.4288
Other				
- Social campaigns	1 (0.2%)	0 (0.0%)	1 (0.3%)	0.5185
- I'm not interested	1 (0.2%)	1 (0.7%)	0 (0.0%)	0.1206

Values express counts (n) and percentages (%).Statistically significant differences between the groups of the two recruitment periods were analysed using the Pearson's chi-square (χ2).
